# Origin, prospective identification, and function of circulating endothelial colony-forming cells in mice and humans

**DOI:** 10.1172/jci.insight.164781

**Published:** 2023-03-08

**Authors:** Yang Lin, Kimihiko Banno, Chang-Hyun Gil, Jered Myslinski, Takashi Hato, William C. Shelley, Hongyu Gao, Xiaoling Xuei, Yunlong Liu, David P. Basile, Momoko Yoshimoto, Nutan Prasain, Stefan P. Tarnawsky, Ralf H. Adams, Katsuhiko Naruse, Junko Yoshida, Michael P. Murphy, Kyoji Horie, Mervin C. Yoder

**Affiliations:** 1 Department of Pediatrics, Indiana University School of Medicine, Indianapolis, Indiana, USA.; 2 Division of Regenerative Medicine, Hartman Institute for Therapeutic Organ Regeneration, Department of Medicine, Weill Cornell Medicine, New York, New York, USA.; 3 Department of Physiology II, Nara Medical University, Kashihara, Nara, Japan.; 4 Department of Surgery,; 5 Department of Medicine,; 6 Department of Medical and Molecular Genetics, and; 7 Department of Anatomy Cell Biology and Physiology, Indiana University School of Medicine, Indianapolis, Indiana, USA.; 8 Center for Stem Cell and Regenerative Medicine, Brown Foundation Institute of Molecular Medicine, University of Texas Health Science Center at Houston, Houston, Texas, USA.; 9 Max Planck Institute for Molecular Biomedicine, Muenster, Germany.; 10 Department of Obstetrics & Gynecology, Nara Medical University, Kashihara, Nara, Japan.; 11 Indiana Center for Regenerative Medicine and Engineering, Indiana University School of Medicine, Indianapolis, Indiana, USA.

**Keywords:** Vascular Biology, Endothelial cells

## Abstract

Most circulating endothelial cells are apoptotic, but rare circulating endothelial colony-forming cells (C-ECFCs), also known as blood outgrowth endothelial cells, with proliferative and vasculogenic activity can be cultured; however, the origin and naive function of these C-ECFCs remains obscure. Herein, detailed lineage tracing revealed murine C-ECFCs emerged in the early postnatal period, displayed high vasculogenic potential with enriched frequency of clonal proliferative cells compared with tissue-resident ECFCs, and were not committed to or derived from the BM hematopoietic system but from tissue-resident ECFCs. In humans, C-ECFCs were present in the CD34^bright^ cord blood mononuclear subset, possessed proliferative potential and in vivo vasculogenic function in a naive or cultured state, and displayed a single cell transcriptome sharing some umbilical venous endothelial cell features, such as a higher protein C receptor and extracellular matrix gene expression. This study provides an advance for the field by identifying the origin, naive function, and antigens to prospectively isolate C-ECFCs for translational studies.

## Graphical abstract

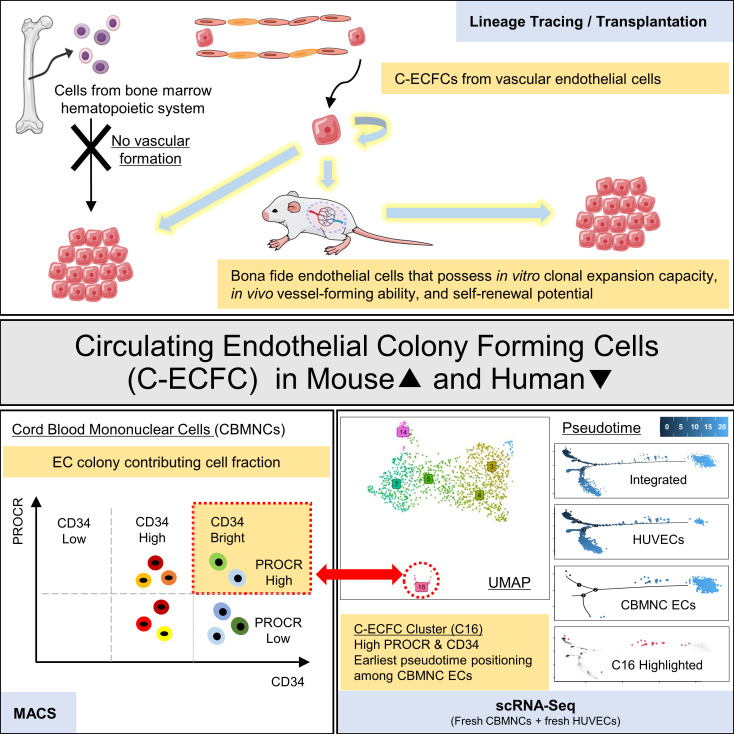

## Introduction

Circulating endothelial cells (C-ECs) are dead or dying ECs sloughed off tissue vasculature (1, 2), and their number serves as a biomarker for various disease states (3, 4) (Table 1, “List of nonstandard abbreviations”). In humans, circulating endothelial colony-forming cells (C-ECFCs), also known as blood outgrowth endothelial cells, with high clonal proliferative potential are a subset within C-ECs in umbilical cord blood (CB) or adult blood (5–8). C-ECFCs are vasculogenic cells displaying in vivo vessel-forming ability, discriminating their functions from BM-derived endothelial progenitor cells (EPCs) (9) restricted to playing proangiogenic roles (10–12). However, several important unaddressed questions remain: Where do C-ECFCs originate, and do C-ECFCs in their in vivo naive state function similarly to isolated, growth factor–expanded, and cultured ECFCs?

We have addressed these questions in mice and found murine C-ECFCs in a developmentally regulated window across several mouse strains. We identified lineage-traced C-ECFCs with high proliferative potential that upon transplantation in a naive or cultured expanded state form robust vasculature. Moreover, single-cell RNA-Seq analysis (scRNA-Seq) of CB distinguished various C-EC subsets. Markers defining the subsets permitted prospective isolation of C-ECFCs from CB with evidence that naive or cultured C-ECFCs display clonal proliferative potential and in vivo vessel formation, defining these cells as a distinct subpopulation of C-ECs. We have thus uncovered key characteristics of C-ECFCs in mice and humans, with information to accelerate C-ECFCs as a viable choice for cell therapy.

## Results

### C-ECFCs in mice possess vasculogenic potential similar to humans.

**Figure 1:**
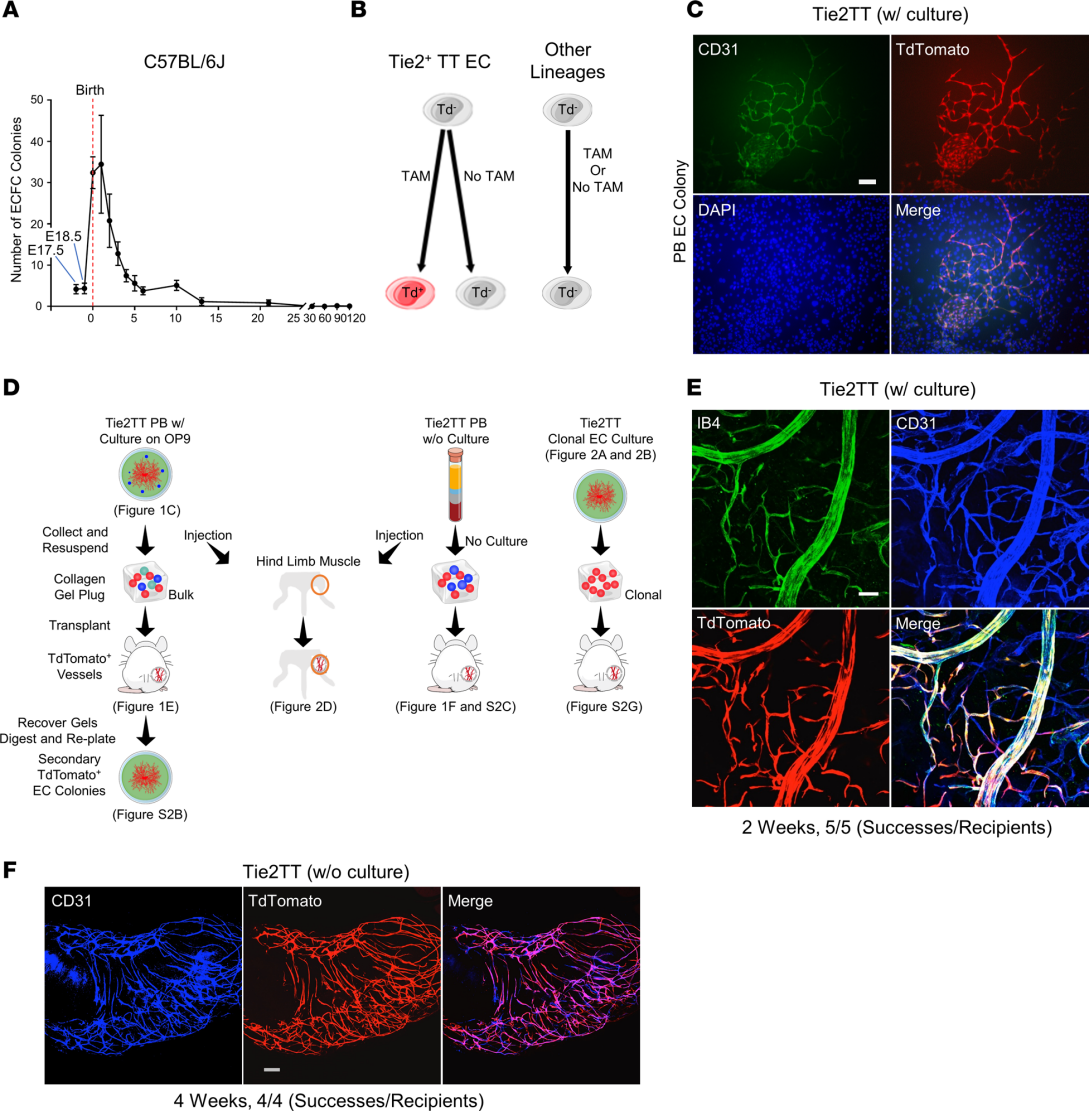
Some murine circulating ECFCs form functional blood vessels in vivo and have the ability to self-renew. (**A**) Kinetics of emergence of ECFCs in C57BL/6J mouse blood; 3–6 mice per time point. (**B**) Schematics of lineage tracing using Tie2TT mice. (**C**) Representative TT^+^ EC colonies derived from PB of Tie2TT mice (155 TT^+^ EC colonies out of 177 colonies from 15 pups). (**D**) Schematic of collagen plug transplantation (all) and cell injection to hind limb muscle (left and middle) using PB-derived cells (left), PB without OP9 culture (middle), and EC colony from clonal EC culture (right) of Tie2TT mouse (P2). TT^+^ vessels can be digested and replated on OP9, resulting in secondary TT^+^ EC colonies (left). (**E**) PB-derived (with in vitro OP9 coculture) TT^+^ vessels are inosculated with host vasculature 2 weeks after transplantation (shown by systemic IB4 i.v. injection); 5 successes out of 5 recipients. (**F**) Uncultured CEC-derived blood vessels (TT^+^) are shown 4 weeks after collagen plug transplantation using PB of Tie2TT (P2); 4 successes out of 4 recipients. Scale bars: 200 μm in **C**, **E**, and **F**. CEC, circulating endothelial cells.

C-ECFCs, though present in humans, are not demonstrable in individual adult mice. Previously, blood from 5–10 mice (>8-week-old C57BL/6J animals) was pooled into a single aliquot and plated in culture and in 5 of 44 (11%) attempts (total 282 mice) was reportedly successful in growing at least 1 C-ECFC colony (13). Since CB ECFCs are enriched 60- to 100-fold compared with adult peripheral blood (PB) (6), we isolated murine PBMCs from prenatal animals to 3 months of age and seeded mononuclear cells (MCs) on OP9 feeder cells (Supplemental Figure 1A; supplemental material available online with this article; https://doi.org/10.1172/jci.insight.164781DS1). This feeder layer supports EC proliferation into spindle-shaped network-type colonies, and we recently found that murine C-ECFCs are effectively and reproducibly cultured with this method across the life span (14, 15). Within 4 days, typical EC colonies appeared from blood of multiple strains (Supplemental Figure 1, B–G), identifying C-ECFCs in each mouse strain. Of interest, the duration of C-ECFCs varied by strain, but overall, the duration of C-ECFC recovery was notably short, with detectable but negligible levels prior to birth, peaking at birth, and rapidly declining in a strain-dependent manner within weeks to months (Figure 1A and Supplemental Figure 1H).

To trace C-ECFCs, we generated a Tie2CreERT2:Rosa-TdTomato (Tie2TT) mouse, which conditionally expresses TT under control of a Tie2 promoter confirmed to be endothelial lineage restricted (16) (Figure 1B). We confirmed the hematopoietic cell (HC) fraction of tissues from these mice showed a negligible expression of TT (Supplemental Figure 2A). Neonatal mice (P0–P3) were treated with tamoxifen (TAM), and P4 PBMCs were plated on OP9 cells. TT^+^ EC colonies were efficiently formed in 155 of 177 (88%) wells (Figure 1C). Recovered PB-derived TT^+^ EC colonies with rare surviving TT^−^ HCs were suspended in a collagen gel plug and implanted s.c. in NOD/SCID mice. Only TT^+^ vessel formation was observed in all gels after 2 weeks (Figure 1, D and E). This result demonstrated that only donor-derived TT vessels were formed in the gels and is consistent with previous published work that cell-free type 1 porcine collagen gels (with added growth factors) alone are not capable of attracting endogenous murine EC-forming vessels (8, 17). Importantly, the donor TT^+^ vessels inosculated with host vessels within 2 weeks of implantation and were perfused with the Isolectin B4 (IB4) i.v. infused (Figure 1E). After cells from vessels were recovered with enzymatic digestion and replated on fresh OP9 cells, secondary TT^+^ EC colonies were identified from every plug tested (Supplemental Figure 2B). Thus, most C-ECFCs can be labeled with the Tie2TT tracing system and possess prominent clonal proliferation and functional in vivo vessel-forming potential.

### Murine and human naive C-ECFCs form functional blood vessels and display ability to self-renew.

**Figure 2:**
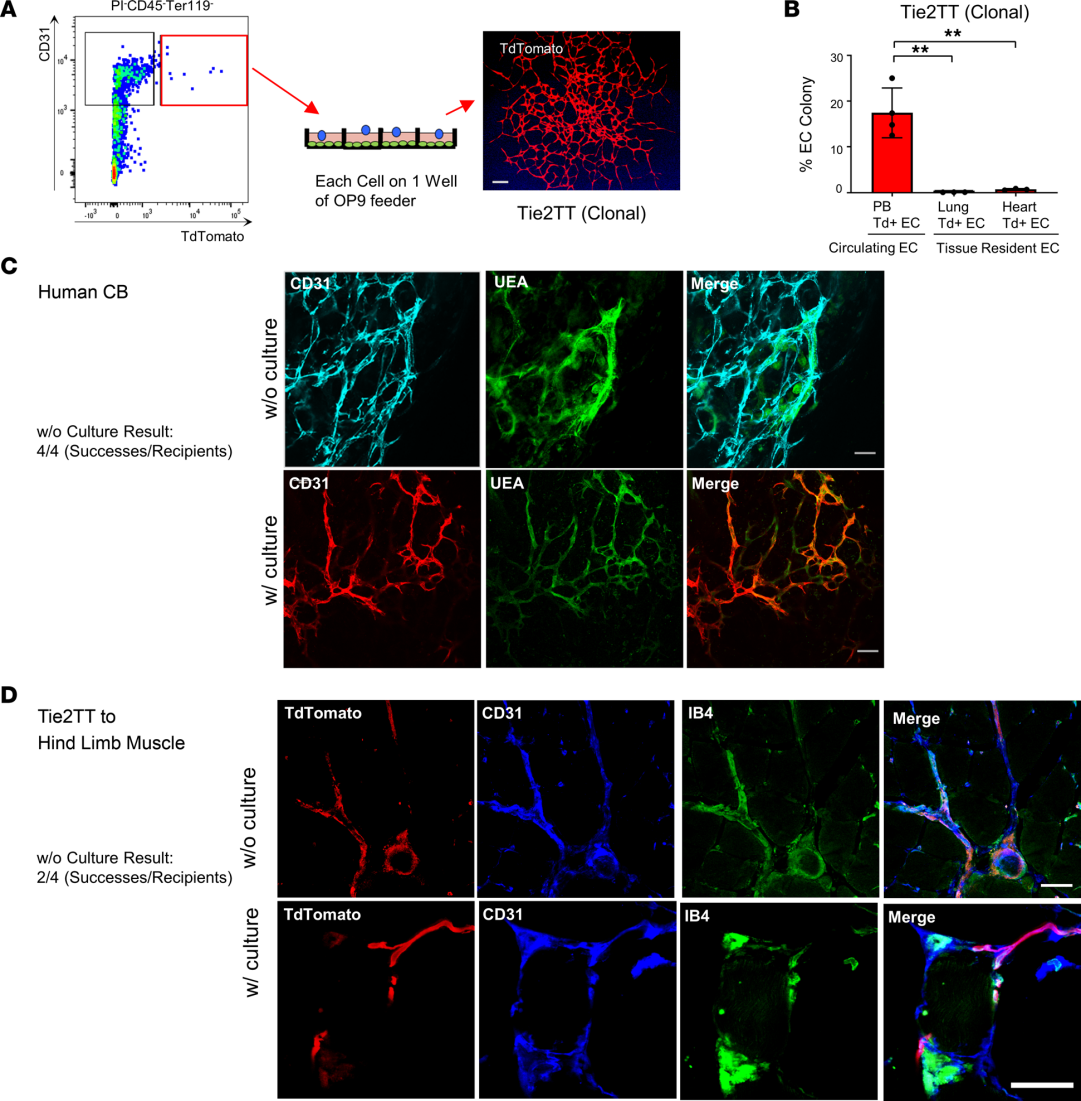
Abundance of murine C-ECFCs with clonal proliferative potential and their vessel-forming capacities with and without prior culture, comparable to human C-ECFCs. (**A**) Schematic of single-cell colony-forming assay using CD45^–^Ter119^–^CD31^+^TT^+^ cells in Tie2TT mice (P2). Right panel shows a representative picture of an EC colony. TT^+^ EC colonies were confirmed at least 4 times. (**B**) Quantitation of the frequency of ECFCs from PB, lung, or heart-derived CD45^–^Ter119^–^CD31^+^TT^+^ cells in Tie2CreTT mice (P2). *n* = 3–4. Data are shown as the mean ± SD. **P < 0.01. Tukey-Kramer post hoc test. (**C**) Uncultured and cultured human CB CD34^+^CD45^–^ cells (MACS sorted) can form functional blood vessels in vivo after transplantation; 4 successes out of 4 recipients of uncultured cells. (**D**) Representative TT^+^ vessel of uncultured and cultured Tie2TT PB injection to hind limb muscle; 2 successes out of 4 recipients of uncultured cells. Scale bars: 200 μm (**C**), 50 μm (**D**).

C-ECFCs’ vasculogenic potential was subsequently tested without any in vitro cell culture step to address any criticism that cultured ECFCs are an artifact of tissue culture adaptation (Figure 1D). Freshly isolated Tie2TT PBMCs were recovered without further enrichment or culture, and the entire dose of unmanipulated PBMCs suspended in the collagen gels formed TT^+^ vessels when implanted in mice (Figure 1F and Supplemental Figure 2C), reflecting the inherent (noninduced) vasculogenic nature of naive C-ECFCs present in circulating murine blood. Next, to assess the clonal proliferative properties, Tie2TT^+^ cells (TAM at P0–P1) in PBMCs and lung and heart ECs were plated at a single-cell level (at P2) (Figure 1D and Figure 2A). EC colonies formed in 17% of wells from PBMCs, a more than 20 times greater frequency than lung or heart ECs (Figure 2B and Supplemental Figure 2, D and E), suggesting postnatal blood is enriched with C-ECFCs. However, tissue Tie2TT^+^ vessel-derived ECFCs possessed clonal progeny with proliferative potential that was greater (>10,000 cells/colony) than C-ECFCs, where the most proliferative clones produced 2,001–10,000 cells (Supplemental Figure 2, D and F). These results suggest C-ECFCs are derivatives of tissue ECFCs since the known proliferative hierarchy of ECFCs dictates that the most proliferative ECFCs give rise to clones restricted to fewer cells/colony (Supplemental Figure 2F) (6, 18).

EC colonies formed from single-plated TT^+^ ECs were recovered, suspended in collagen, and s.c. implanted, where implants are totally dependent upon formation of vasculature and connection to host vessels to sustain implanted EC survival in the ischemic environment (8), and TT^+^ vessels were identified and survived for up to 4 months (Supplemental Figure 2G). This indicates some single Tie2^+^TT C-ECFCs display remarkable clonal proliferative potential, in vivo vessel forming potential, and evidence that C-ECFCs are derivatives of resident tissue Tie2^+^TT ECFCs.

Similar to C-ECFCs in postnatal mice, human CB ECFCs form a hierarchy of clonal EC colonies and display vasculogenesis in vivo (6, 18). We now report that freshly isolated CD34^+^CD45^−^ CBMCs exhibit vasculogenesis in all gels after transplantation without prior in vitro culture comparable to CB ECFCs isolated and expanded in culture (Figure 2C). Moreover, cells recovered from the formed vessels give rise to secondary ECFC colonies upon replating (Supplemental Figure 2H). Thus, circulating CB ECFCs form vessels in vivo, which by nature hold endothelial intima in quiescence by endocrine, autocrine, paracrine, and mechanical inputs (19), but within which self-renewing ECFCs were maintained that produced secondary ECFCs upon plating in vitro.

To demonstrate whether naive murine C-ECFCs contribute to tissue vasculature in murine tissue, we injected PB (pooled blood of 6 neonates, P2) of Tie2TT mice (TAM at P0–P1) directly into the hind limb muscle of host C57BL/6J mice. TT^+^ EC engraftment was confirmed in the tissue injected with PB itself (2/4 recipient mice) including evidence that the vessels were functionally active, inosculated to host vessels, and perfused with systemically injected IB4 lectin (Figure 2D). These findings highlight naive C-ECFCs directly contribute to vascular formation in murine tissues in vivo.

### Murine C-ECFCs are derived from resident ECs.

**Figure 3:**
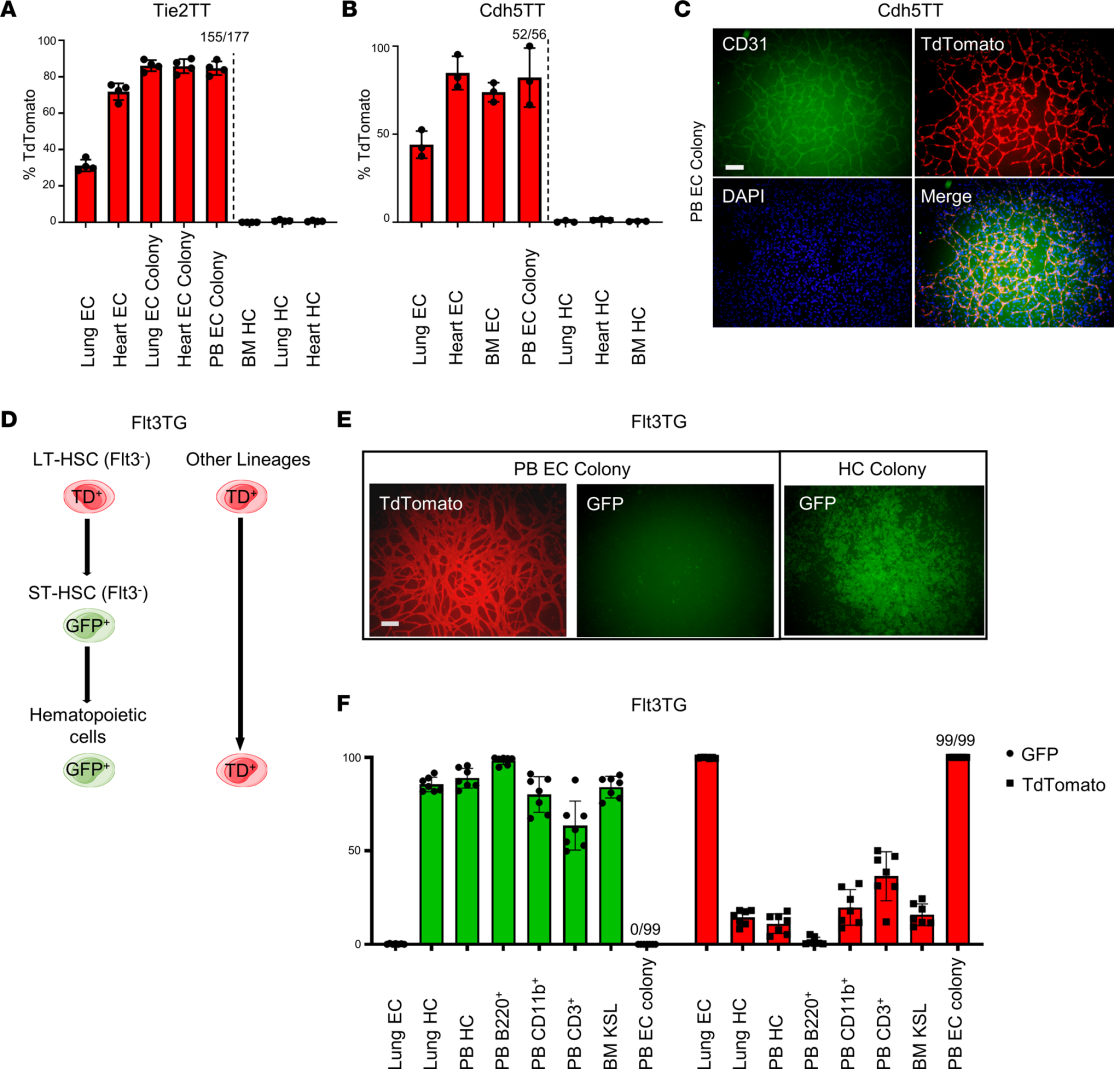
Murine C-ECFCs are derived from resident ECs. (**A** and **B**) Quantitation of labeling efficiency of endothelial and hematopoietic lineage, and resultant EC colonies in each tissue/organ from Tie2TT and Cdh5TT mice. Data are shown as the mean ± SD; colonies were counted in at least 3–4 independent experiments. (**C**) Representative TT^+^ EC colonies derived from PB of Cdh5TT mice (52 TT^+^ EC colonies out of 56 colonies, see also **B**). (**D**) Schematics of lineage tracing using hematopoietic specific Flt3TG mice. (**E**) Representative TT^+^ EC colonies (left) and GFP^+^ HC colonies (right) derived from PB of Flt3TG mice (99 TT^+^ EC colonies out of 99 colonies, see also **F**). (**F**) Percentage of GFP- and TT-expressing cells in the fraction of lung EC, lung HC, BM KSL, PB HC, PB B220^+^, PB CD11b^+^, and PB CD3^+^. All EC colonies derived from peripheral MCs are TT^+^. Data are shown as the mean ± SD; GFP or TT expression was evaluated in at least 7 independent experiments. Scale bars: 200 μm. KSL, c-Kit^+^Sca1^+^Lineage^–^ cell.

While TT specifically labeled ECs, but not HCs, in organs (Figure 3A and Supplemental Figure 2A), we asked whether other vascular endothelial or blood cell restricted reporters marked C-ECFCs. Cdh5TT mice conditionally express TT under control of the endothelial-specific Cdh5 promoter (20). Most tissue-resident ECs were TT^+^ but HCs were TT^–^ (Figure 3B). Culturing PB from Cdh5TT mice on OP9 cells led to identification of TT^+^ EC colonies in 52/56 (93%) of the wells (Figure 3, B and C), suggesting C-ECFCs are derived from vascular ECFCs that constitutively express either Tie2 or Cdh5. Indeed, such behavior of isolated resident ECFCs is similar to resident vascular endothelial stem/progenitors (VESPs) with ECFCs potentially known to reside within the vascular endothelial intima and shown to play a major role in vascular regeneration (21, 22).

Next, we confirmed the endothelial origin for C-ECFCs and not a BM hematopoietic origin, as claimed for EPCs (9), using the hematopoietic restricted Flt3cre mTmG reporter mouse (23) (Figure 3D and Supplemental Figure 3A). In this animal, TT is ubiquitously expressed in all cells, except those cells that express Flt3cre, since Cre-mediated recombination irreversibly switches TT to GFP fluorescence. Consistent with prior work, flow cytometric analysis identified Flt3^+^ short-term hematopoietic stem cells (HSCs) and all of their hematopoietic progeny to express GFP (Figure 3, D and F, and Supplemental Figure 3B) (24). As anticipated, in vitro methylcellulose- and growth factor–stimulated HC colonies from Flt3cre mTmG PBMCs were labeled with GFP (Figure 3E). The MCs isolated from 25 individual Flt3cre mTmG mice formed a total of 99 EC TT^+^ colonies (PB EC colony), and none expressed GFP (Figure 3, E and F), similar to control primary lung ECs. Thus, C-ECFCs are not committed to or derived from the BM hematopoietic lineage but are marked by typical endothelial transgenic markers.

### scRNA-Seq reveals EC-related clusters in circulating human CBMCs.

**Figure 4:**
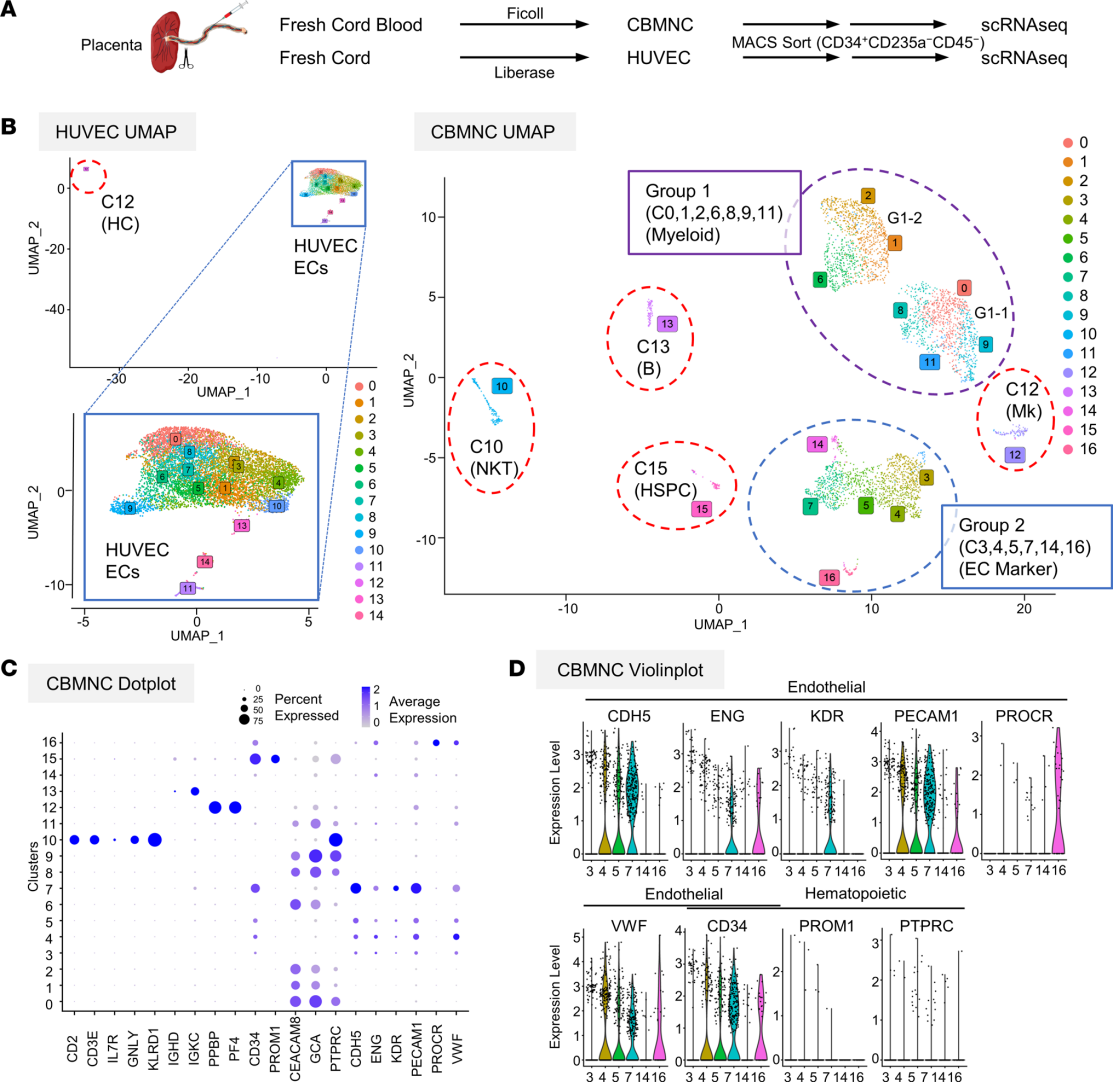
scRNA-Seq reveals EC-related clusters in circulating CBMCs. (**A**) Schematic of scRNA-Seq assay using freshly isolated CBMCs and HUVECs, using Ficoll density centrifugation or liberase enzymatic digestion, respectively. CD34^+^CD235a^−^CD45^−^ cells are enriched by MACS. (**B** and **C**) Global UMAP plots of CBMCs and HUVECs and dot plots of scaled average expression of major canonical markers (columns) in all clusters of CBMCs (rows). (**D**) Violin plots of EC genes comparing EC marker-expressing clusters of CBMCs.

**Figure 5:**
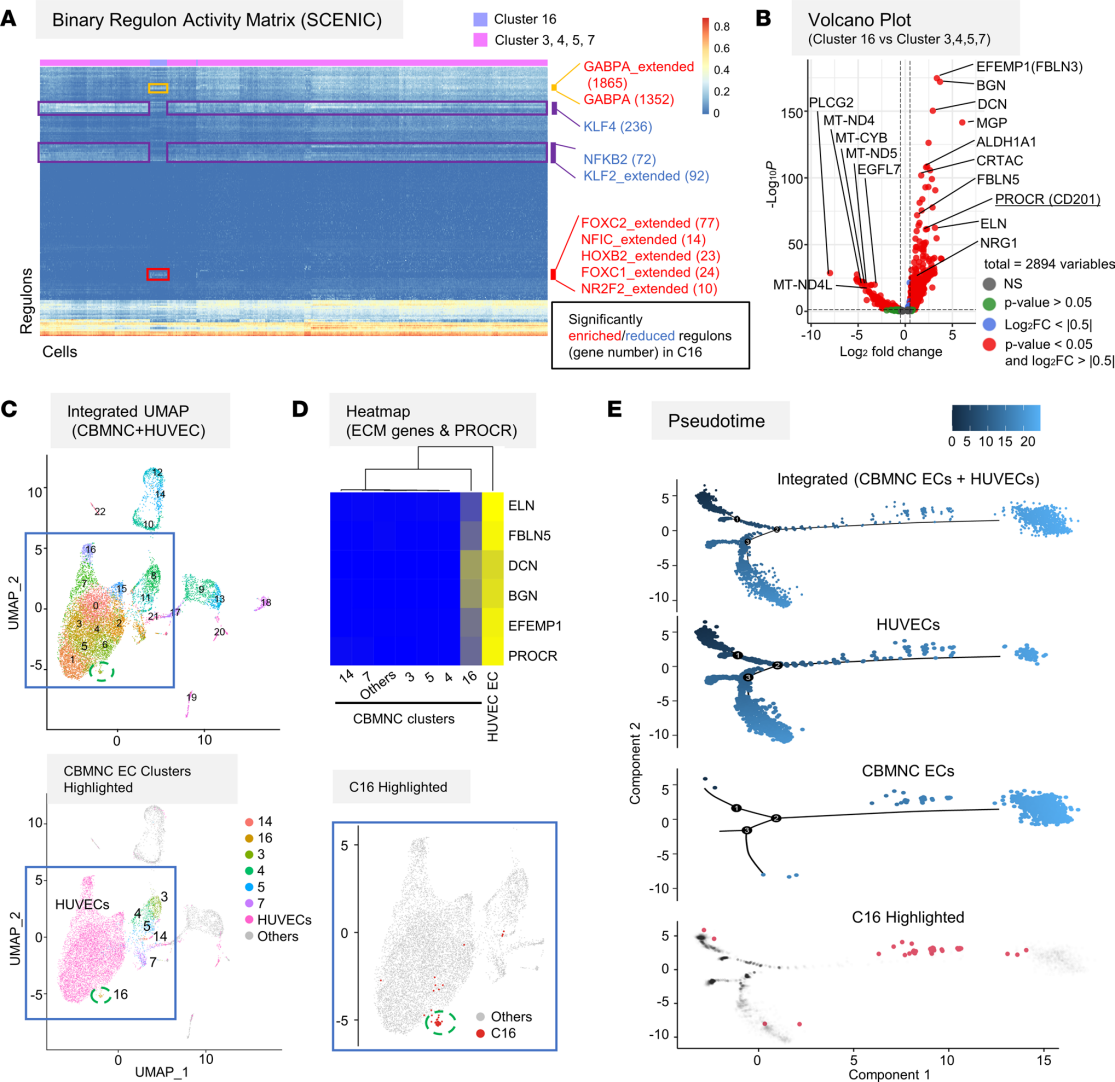
C16 is a candidate cluster enriched for C-ECFC population characterized by PROCR expression and may be derived from resident vascular ECs. (**A**) SCENIC analysis result on CBMC clusters. Binary regulon activity matrix is shown with AUCell scores, indicating the activity of each TF regulon in each cell. Some significantly enriched or reduced TF regulons in C16 are shown at right (with related gene number). (**B**) Volcano plots comparing C16 versus C3, C4, C5, and C7. Representative genes are shown. PROCR is the only cell surface marker gene in the plot with a significant *P* value. (**C**) Integrated UMAP of CBMCs and HUVECs (left above). CBMC EC clusters and C16 highlighted (left below and right below), respectively. (**D**) Heatmap of mean expression of ECM genes and PROCR in annotated clusters. (**E**) Pseudotime analysis using CBMC ECs (C3, C4, C5, C7, C14, and C16) and HUVECs (w/o C12), showing integrated plot (top) and sample plots (2 in the middle). C16 (red) is highlighted below.

To discriminate human C-ECFCs from other blood cells, we compared scRNA-Seq of fresh CBMCs and HUVECs isolated from matching umbilical cords (Figure 4, A and B). We annotated hematopoietic lineage clusters (group 1-1 and 1-2, myeloid; cluster 10 [C10], NK and T; C12, Mk; C13, B; and C15, hematopoietic stem/progenitor cell [HSPC]) based on characteristic genes (Figure 4, A and C, and Supplemental Figure 4A) (25, 26). A second major cell population (group 2) consisted of 6 clusters (C3, C4, C5, C7, C14, and C16) expressing EC markers such as PECAM1, CDH5, and VWF, but not HC genes (Figure 4, B–D). SCENIC analysis (27) identified FOXC2, FOXC1, and NR2F2 transcriptional factor (TF) regulons as significantly enriched in C16 (Figure 5A and Supplemental Table 1). C3–5 and C7 showed significant enrichment of KLF2, KLF4, and NFKB2 regulons, indicative of response to blood shear stress (Figure 5A) (28). In C14, most EC-related TF regulons were repressed (Supplemental Table 1), and higher globin and glycophorin expression were observed (Supplemental Figure 4B), suggesting C14 may represent residual rare hemangioblastic ECs present during early pregnancy of placental origin (29). Such results suggest C16 as a candidate of C-ECFCs; an early differentiated cluster with enriched expression of TFs of endothelial development, moderate expression of typical EC markers, and increased protein C receptor (PROCR) expression, previously purported as a marker of murine vascular ECs with in vivo vessel forming potential (Figure 4D) (30, 31). Volcano plots depict the enrichment of ALDH1A1, known to be expressed in prehematopoietic hemogenic ECs (32) and extracellular matrix (ECM) genes, such as EFEMP1, BGN, and DCN (Figure 5B). To further investigate the relationship between C16 and HUVECs, we integrated CBMCs and HUVEC uniform manifold approximation and projections (UMAPs) (Figure 5C). C16 cells were located closest to or inside the large cluster groups created by HUVECs (41/45 cells) with the majority of C16 cells forming a cluster with HUVECs (25/41 cells) (Figure 5C). Notably, while ECM genes and PROCR were expressed highly in HUVECs, only C16 cells among CBMCs retained their expression, albeit at a reduced level (Figure 5D). Finally, the trajectory analysis of integrated CBMC ECs and HUVECs showed HUVECs were present at earlier times than most CBMC ECs, and C16 was clearly shown to be in the transitional path between the two (Figure 5E). Taken together, these results suggest C16 (C-ECFC candidate) displays transcriptome features resembling HUVECs, more than any other CBMC clusters and trajectory evidence for derivation from resident vascular ECs.

### Colony-forming potential of PROCR^hi^ and CD34^bright^ endothelial population in CBMCs implicates the existence of C-ECFCs.

**Figure 6:**
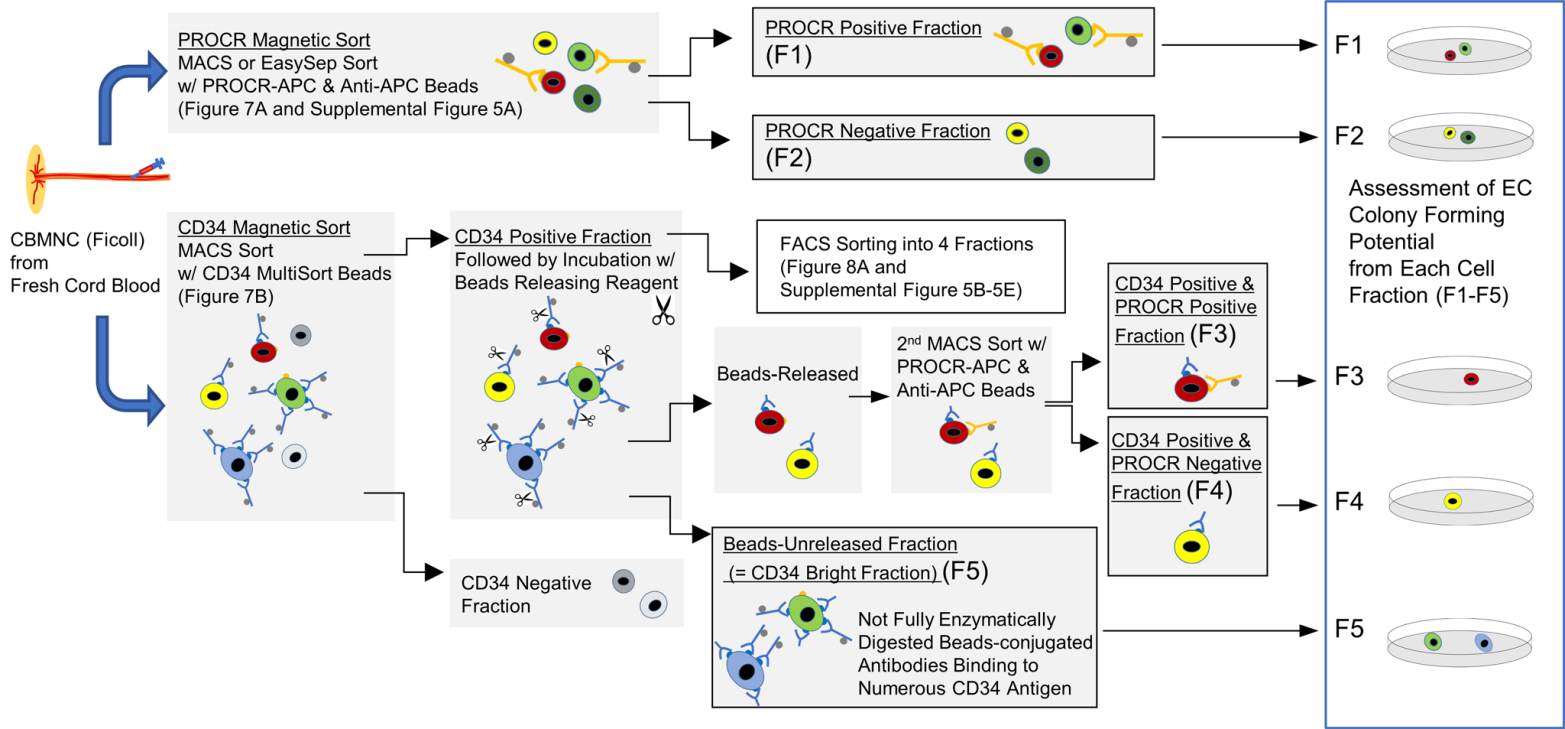
Schematic of in vitro EC colony-forming assay using fresh CB. After Ficoll separation, cells were magnetically sorted with PROCR-APC and anti-APC beads, followed by culture using cells from each fraction: (F1) and (F2). Below: Cells were magnetically sorted with CD34 MultiSort Beads and CD34^+^ fraction then incubated with beads-releasing reagent. The beads-released CD34^+^ fraction was further sorted with PROCR-APC and anti-APC beads, leading to (F3) and (F4), and followed by placement in culture. Beads-unreleased CD34^+^ fraction (enriched with CD34^bright^ population) (F5) was also cultured.

**Figure 7:**
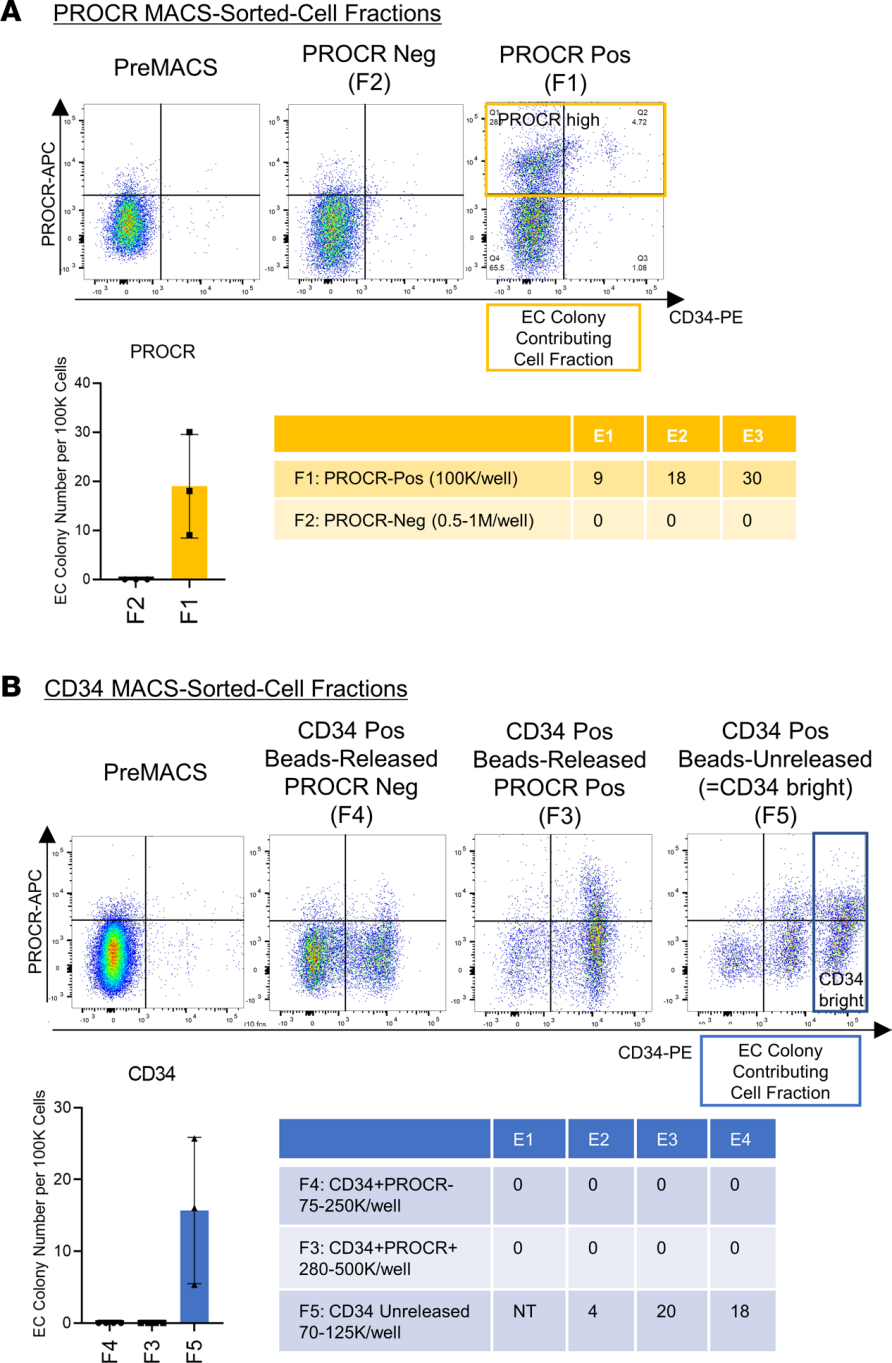
Colony-forming potential of PROCR^hi^ and CD34^bright^ endothelial population in CBMC identifies C-ECFCs within CECs. (**A** and **B**) Flow cytometry analysis of pre- and post-MACS for each fraction (F1–F2 in **A**, F3–F5 in **B**). Quantitation of the number of EC colonies per 100K cells (graph). Table (above, yellow; below, blue) shows seeding cell fraction (with cell number per well) and EC colony number in each experiment. E, experiment; NT, not tested.

**Figure 8:**
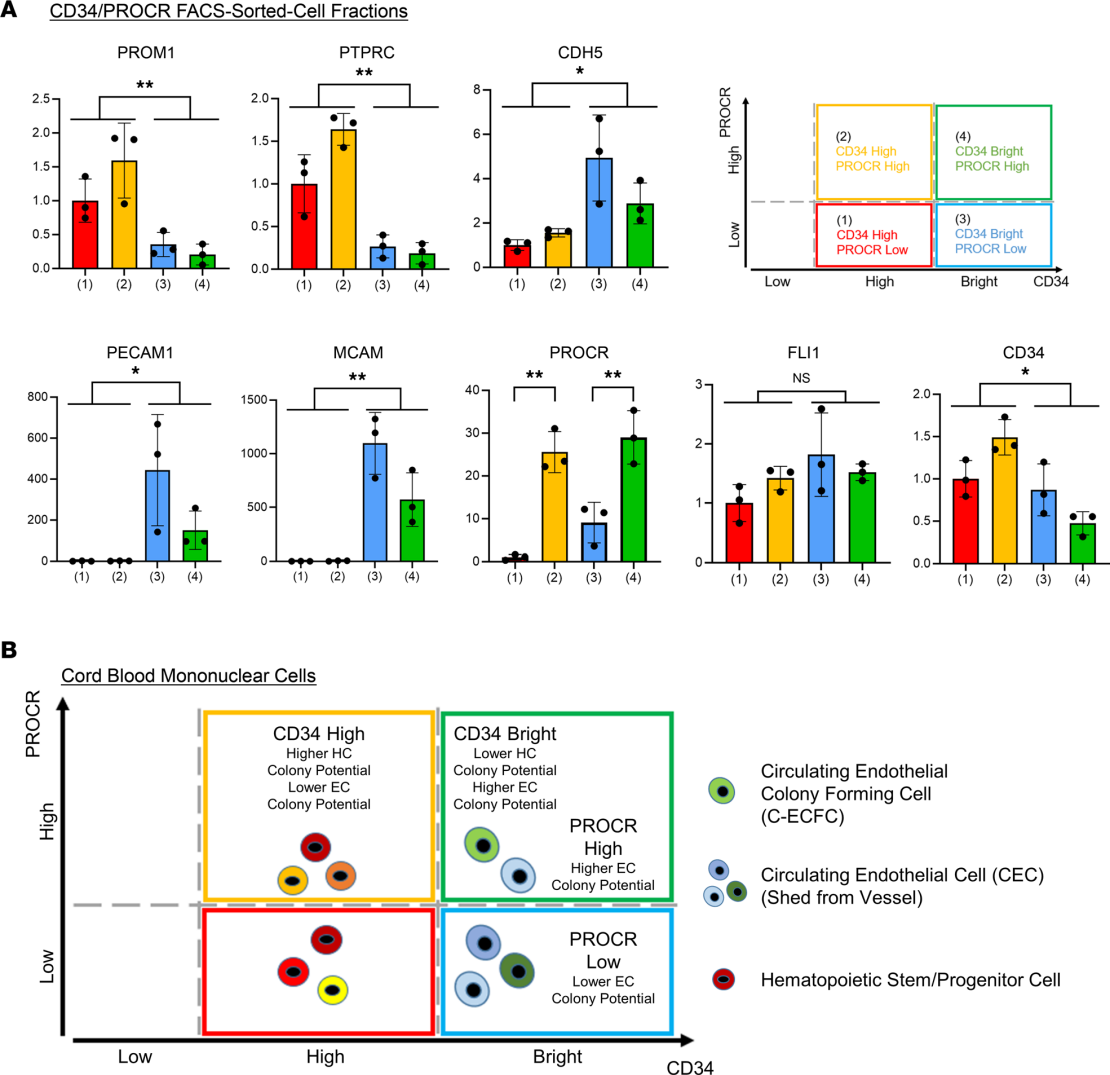
Characterization of PROCR^hi^ and CD34^bright^ cells and overall schematic of human CBMCs classified by PROCR and CD34 expression. (**A**) Fold changes of the expression of EC- and HC-related genes in CD34^bright/hi^ with PROCR^hi/lo^ fractions after FACS. Schematic of each fraction — (i) CD34^hi^ and PROCR^lo^, (ii) CD34^hi^ and PROCR^hi^, (iii) CD34^bright^ and PROCR^lo^, and (iv) CD34^bright^ and PROCR^hi^ — in flow cytometry (right above). Data are shown as the mean ± SD. *n* = 3. Student’s *t* tests were used to compare 2 groups — CD34^hi^ (i) and (ii) versus CD34^bright^ (iii) and (iv). Tukey-Kramer post hoc test was used to compare multiple groups in PROCR expression. *P < 0.05; **P < 0.01. (**B**) Schematic of presumed roles from each fraction using CD34 and PROCR expression in flow cytometry based on the results of the EC colony contributing cell fraction from Figure 7A (yellow dots) and Figure 7B (blue dots).

Confirmation of scRNA-Seq results using colony assays demonstrated the appearance of EC colonies only from PROCR-enriched fraction (Figure 6, Figure 7A, Supplemental Figure 5A, and Supplemental Table 2), indicating PROCR is a potentially novel marker for C-ECFCs. The capacity of CD34^+^ to identify C-ECFCs has been reported by several groups (18, 33), and herein we have demonstrated uncultured naive CD34^+^ populations form vessels, like cultured cells, in vivo (Figure 2C). Magnetic-activated cell sorting (MACS) selected CD34^+^ fractions identified CD34^bright^ cells in all CB samples (*n* = 17) (Supplemental Figure 5B). Recently, C-ECs have been reported to be present in the CD34^bright^ population, but not in the CD34^hi^ or CD133^+^ population (34–36). Here, we discovered a method to magnetically enrich the CD34^bright^ population (Figure 6 and Figure 7B). The highest CD34-expressing cells failed to be released from the magnetic beads after incubating the cells for the manufacturer’s instructed time, and we discovered that C-ECFCs only emerged from this CD34 beads-unreleased fraction; the CD34^bright^ enriched population (Figure 7B and Supplemental Table 2). Since PROCR expression presents in CD34^high^ and CD34^bright^ subsets, functional properties of the 4 fractions were tested following FACS isolation (Supplemental Figure 5B). A smaller subset of the CD34^bright^ population formed HC colonies (Supplemental Figure 5C) compared with CD34^hi^ fraction. In contrast, EC colonies were observed only in the CD34^bright^PROCR^hi^ fraction (1 out of 3) and, otherwise, no colonies were observed (Supplemental Figure 5, D and E), consistent with the known blunting of ECFC colony formation encountered by FACS (18). As supporting evidence, quantitative gene expression analysis revealed significantly lower PROM1 and PTPRC and higher EC marker expression in CD34^bright^ cells. CDH5, PECAM1, and MCAM expression was lower in the PROCR^hi^ fraction, suggesting this fraction may be more immature (Figure 8A). In sum, our studies showed the CD34^bright^ population is representative of C-ECs, of which the PROCR^hi^ subset displays features of C-ECFCs that possess clonal proliferative potential (Figure 8B).

## Discussion

We report that murine C-ECFCs display evidence of emergence from tissue-resident vessel ECs, in vivo vessel-forming potential in a naive or clonal expanded state, clonal proliferative potential, and ECFC self-renewal from recovered implanted vessels in host mice. One might be tempted to conclude identification of a circulating endothelial stem/progenitor cell state, as has been reported for VESPs (21, 22, 30). However, such a claim must prove that putative endothelial stem/progenitor cells can become arteries, veins, capillaries, and lymph vessels and that the cells can repopulate these diverse vascular states after injury (37). Future work will be required to prove murine C-ECFCs possess endothelial stem/progenitor potency.

The negligible number of C-ECFCs at E18.5 and their sudden appearance at P0 (Figure 1A) suggests that emergence of TT^+^ colonies in the blood at birth is unlikely to have originated from the rare preexisting C-ECFCs that appear at E9.5 (15) and decline at E18.5 (Figure 1A); such a burst in circulating cells would require an approximately 8-fold increase in less than 36 hours. Given the greater proliferative potential of emerging clones of resident vascular ECFCs (Supplemental Figure 2, D–F), it is more plausible that they reside upstream in the ECFC hierarchy and would give rise to C-ECFCs.

The molecular mechanisms that may mobilize C-ECFCs into the blood at birth remain undetermined. Some of the most dramatic changes in human physiology occur at the time of birth, particularly in the cardiorespiratory system (38). Survival in utero depends upon circulation of blood through the low resistance placental vascular network and oxygen tension in fetal blood is by necessity low (partial pressure of oxygen in umbilical venous blood; 30 mmHg). With birth, clamping of umbilical cord vessels and removal of placenta, and the taking of the first breath by the baby, lead to the first bolus of air (oxygen is a principal vasodilator) filling the previously airless lung, tethering open the pulmonary vascular bed, and there is an abrupt decline in pulmonary vascular resistance. The pulmonary circulatory vasodilators such as nitric oxide, prostacyclin, prostaglandin D_2_, and bradykinin all work with the increased oxygen concentration to dramatically increase pulmonary blood flow and raise left atrial and ventricular outputs that in time initiate closure of the ductus arteriosus and foramen ovale (39). Concomitantly, there is a surge of cortisol, triiodothyronine, adrenaline, and noradrenaline concentrations in newborn infant blood that is attributable to birth stress (40, 41). While such stressors are known to mobilize WBCs (42), including HSCs (43), into the newborn circulation, further studies will be required to assess the individual or combinatorial roles played by all these physiologic perturbations associated with birth on C-ECFC mobilization.

We provide clear evidence for naive C-ECFC possession of in vivo vessel-forming potential in mice and humans. These data resolve any lingering challenges that naive C-ECFCs lack vasculogenic activity unless isolated and expanded in culture with added endothelial growth factors. Likewise, lineage tracing results with Flt3cre mTmG mice have clarified C-ECFCs are not derived from BM HCs as originally predicted (9).

ScRNA-Seq results have identified several C-EC subsets in human CB (Figure 4, C and D, and Supplemental Figure 4, B–D). Of greatest interest, C16 is enriched with TF regulons for EC early development and ECM genes such as fibulin, decorin, and matrix gla-protein that are critical for cell adhesion (44) and vasculogenesis (45, 46) (Figure 5, A and B). In the integrated UMAP and trajectory analysis (Figure 5, C–E), C16 localized closely with HUVECs, retained upregulated ECM genes, and existed in the transitional region (HUVECs to CBMC ECs) in pseudotime, suggesting this population was derived from resident vascular ECs. Whether C-ECFCs are mobilized from all tissue vasculature and the mechanism for potential mobilization remains unknown, although prior work has identified severe acute myocardial ischemia to induce mobilization of high proliferative potential ECFCs into the blood stream of pigs and humans (47, 48).

We found PROCR as a potentially novel marker for human C-ECFCs. Since PROCR was the only CD marker enriched in C16, we showed that the PROCR^hi^ cell fraction and the CD34^bright^ population are clearly enriched in EC colony formation (Figure 6 and Figure 7, A and B) and conclude the CD34^bright^ and PROCR^hi^ C-ECs as displaying properties of C-ECFCs (Figure 8B). Recently, Procr^+^ endothelial precursors were reported as a candidate tissue resident vascular endothelial stem cells and evidence was presented that these cells play a pivotal role in early fetal vascular development in the mouse (30, 31). These reports are consistent with our results that the PROCR-enriched cluster (C16) located in the earliest pseudotime in the transitional path from HUVECs to CBMCs may represent such a precursor. However, whether human resident vascular ECFCs expressing PROCR display a possible contribution to the hemogenic endothelium (31) has not been tested in our study. Further studies on PROCR^+^ ECs in humans as well as mice are desirable. Taken together, our study provides what we believe to be novel insights that C-ECFCs can be potentially enriched for further studies toward translation to the clinic.

### Limitations of the study.

We did not perform transcriptome studies that can explore the potential marker gene expression for murine C-ECFCs given the small amount of blood that can be obtained from each postnatal mouse. Furthermore, we did not prospectively isolate human C-ECFCs for in vivo vessel-forming studies and we inferred the CD34^bright^PROCR^+^ population is responsible for EC colony formation based on our sequential magnetic isolation results. Finally, we can only deduce that C-ECFCs are mobilized from resident vessel ECFCs through use of several lineage-tracing transgenic approaches in mice and scRNA-Seq in human studies, as we cannot directly image the emergence of C-ECFCs into circulation induced by metabolic or physiologic changes occurring at birth.

## Methods

### Animals.

C57BL/6J (JAX 000664), FVB/NJ (FVB, JAX 001800), 129S1/SvImJ (SV129, JAX 002448), B6.Cg-*Gt(ROSA)26Sor*^tm14(CAG-TT)Hze^/J (49) (R26R-TT, JAX 007914), and NOD.Cg-*Prkdc*^scid^/J (NOD/SCID, JAX 001303) were purchased from the Jackson Laboratory. CD1 mice (022) were purchased from Charles River Laboratories. Flt3Cre^+^ ROSA^mTmG/mTmG^ mice (50–52) were a gift from Slava Epelman, University of Toronto, Toronto, Ontario, Canada. Tie2CreERT2 mice (gift of Ye Zheng, Cincinnati Children’s Hospital Medical Center, University of Cincinnati, Cincinnati, Ohio, USA), previously described (16), were crossed with the commercially available R26R-TT mice (above) to generate (Tie2TT) mice. The primers used for genotyping the abovementioned Cre or ROSA mice are shown in Supplemental Table 3. To induce Cre expression in Tie2CreERT2 and Cdh5(PAC)-CreERT2 mice, 50 mg/kg TAM was injected into the animals i.p. at appropriate time points (3–5 days).

### Murine cell collection.

Blood was collected from postnatal mice by cardiac puncture. After blood collection, MCs were isolated by resuspending in RBC lysis buffer (QIAGEN) for 10 minutes (for culture) or density gradient centrifugation using Histopaque-1083 (Sigma) (for flow cytometry).

To collect cells from mouse lung, liver, or heart, tissues were dissected from euthanized mice and were minced with razor blades. Samples were digested with 0.25% collagenase I (StemCell Technologies) at 37°C for 30 minutes. After digestion, the samples were resuspended in medium, pipetted thoroughly, and passed through 70 μm cell strainers to remove cell clumps. To collect mouse BM cells, tibias and femurs were dissected and cleaned with scissors to remove remaining muscle tissues. Then the bones were crushed with a pestle and mortar before the cells were digested and filtered like the other tissues above.

### Human cell collection.

To collect HUVECs, the umbilical vessels were flushed with PBS 3 times. Then, one end of the vessel was clamped and liberase solution (500 μL stock solution diluted with 24.5 mL PBS; Roche) was infused into the vessel through the open end before it was clamped. The liberase-infused vascular tissues were incubated at 37°C for 14 minutes to release ECs from the basement membrane. Finally, the solution containing digested ECs was flushed into 50 mL tubes for centrifugation and EC recovery.

To collect MCs from human umbilical CB samples, Ficoll-Paque (GE Healthcare) was added to the anticoagulated blood (diluted in PBS) and cell separation was performed according to the manufacturer’s protocol.

### MACS for scRNA-Seq.

CBMCs and HUVECs for scRNA-Seq were MACS sorted with CD45^–^, CD235a^–^, and CD34^–^ MicroBeads (Miltenyi) using the following method. First, freshly isolated CBMCs and HUVECs were resuspended in PBS buffer (PBS with 10% FBS; Hyclone). Then, 10–-20 μL of CD45 MicroBeads (human, 130-045-801) and 10–20 μL CD235a (Glycophorin A) MicroBeads (human, 130-050-501) were added per 1 × 10^7^ total cells and incubated at 4°C for 13 minutes (and tubes were tapped every 3–4 minutes). Cells were washed, centrifuged at 400*g* for 7 minutes, and resuspended 1 × 10^8^ cells in 500 μL of PBS buffer. Cell suspensions were then applied to the LD column (Miltenyi Biotec). Unlabeled cells (CD45^–^CD235a^–^ enriched fraction) that passed through the column were collected and counted for the next step. Collected cells were centrifuged at 400*g* for 5 minutes, resuspended with 60 μL of PBS buffer, and 20 μL of Fc Block reagent and 20 μL CD34 Microbeads (human, 130-046-702) were added and incubated at 4°C for 20 minutes (and tubes were tapped every 3–4 minutes). Cells were washed and centrifuged at 400*g* for 5 minutes and resuspended in 500 μL of PBS buffer. Cell suspensions were then applied to the LS column (Miltenyi Biotec). After washing 3 times, magnetically labeled cell suspensions were immediately flushed out, filtered with a 30 μm filter (Sysmex) and counted. CD45^–^CD235a^–^CD34^+^ enriched CBMCs and HUVECs (scRNA-Seq samples) were then suspended in DMEM (Gibco)/10% FBS (Hyclone) in 500–1,000 cells per 1 μL concentration and used for scRNA-Seq (see below).

### Single-cell library preparation.

The scRNA-Seq samples (freshly isolated and MACS-sorted CBMCs and freshly isolated and MACS-sorted HUVECs from the same individual human) were applied to a single cell master mix with lysis buffer and reverse transcription reagents, following the Chromium Single Cell 3’ Reagent Kits V2 and V3 User Guide (10x Genomics). This was followed by cDNA synthesis and library preparation. All libraries were sequenced in Illumina NovaSeq6000 platform in paired-end mode (28 bp + 91 bp). The total number of CBMCs and HUVECs were 4,477 cells and 13,651 cells, respectively. Next, 85,000 reads per cell were generated and 94% of the sequencing reads reached a *q* score of at least 30 (Q30) in CBMC, while 28K reads per cell were generated and 95% of the sequencing reads reached Q30 in HUVECs.

### Single-cell data processing.

The 10x Genomics Cell Ranger (v. 2.1.0) pipeline was utilized to demultiplex raw base call files to FASTQ files and reads aligned to the human reference genome GRCh38 using RNA-Seq aligner STAR (53). The data set has been deposited in the NCBI’s Gene Expression Omnibus database (GEO GSE220468). Cell Ranger computational output was then analyzed in R (v.3.5.0) using the Seurat package v. 3.0.1 (54). Seurat objects were created for nonintegrated and integrated data using the following filtering metrics: gene counts were set between 200 and 3,000 and mitochondrial gene percentages less than 25 in CBMCs and HUVEC, gene counts were set between 3,500 and 10,000 and mitochondrial gene percentages less than 10 to exclude doublets and poor-quality cells. We then removed ribosomal protein genes to reduce noise of dead and/or dying cells. Gene counts were log transformed and scaled to 0–5. The top 20 principal components were used to perform unsupervised clustering analysis and visualized using UMAP dimensionality reduction (resolution 0.8). Using the Seurat package, annotation and grouping of clusters to cell type were performed manually by inspection of differentially expressed genes for each cluster, based on canonical marker genes in the literature (25, 26).

### Upstream regulatory network analysis and trajectory analysis.

SCENIC analysis (27) was performed using the default setting and hg19-500bp-upstream-7species.mc9nr.feather database was used for data display.

### Pseudotemporal ordering of single cells.

We performed pseudotime analysis on the integrated Seurat object containing all cells in HUVEC clusters without C12 as well as those in EC clusters (C3, C4, C5, C7, C14, and C16). The data sets were analyzed through the R package Monocle using default parameters. Outputs were obtained detailing the pseudotime cell distributions for each cell type (including cells in C16, which were colored in red). Positional information for the monocle plot was used to color cells (55).

### Magnetic enrichment of CD34^bright^ and CD34^hi^ fractions and PROCR^hi^ and PROCR^lo^ fractions.

To enrich PROCR^hi^ and PROCR^lo^ fractions (Figure 7A), freshly isolated CBMCs were magnetically sorted using CD201 (EPCR) Ab (Miltenyi Biotec, clone REA337) and Anti-APC MicroBeads (Miltenyi Biotec, clone 130-090-855) or EasySep APC Positive Selection Kit (StemCell Technologies, catalog ST-18453) according to each manufacturer’s protocol.

To enrich the CD34^hi^PROCR^hi^ fraction, we initially planned to perform a 2-step MACS using CD34 MultiSort MicroBeads (Miltenyi Biotec). The kit can separate CD34 Ab bound to the cell surface from the conjugated magnetic beads by using a proprietary enzyme called “release reagent” after sorting with the Ab beads-tagged cells in the first step. This allows for a second step Ab beads-tagged reaction for isolating PROCR Ab-bound cells. However, we noticed that there were cells in a fraction (unreleased fraction) whose beads could not be separated by the release reagent. This fraction may be caused by the fact that the amount of CD34 antigen on the cell surface is very high (i.e., CD34^bright^); therefore, not all of the Ab bead-tagged cells can be separated in the prescribed release reagent reaction (Figure 6). Therefore, we proceeded to isolate cells in the following manner.

Freshly isolated CBMCs were counted and labeled with 100 μL of Fc block reagent and 100 μL of CD34 MultiSort MicroBeads (human, 130-056-701) per 1 × 10^8^ total cells. Then, the first magnetic separation was performed with LS columns. After magnetically labeled CD34^+^ fraction was flushed out, 20 μL of MultiSort Release Reagent were added per 1 mL of cell suspension and incubated for 10 minutes in the refrigerator in the dark (and tubes were tapped every 3–4 minutes). Next, the second magnetic separation was performed. This column can bind bead-unreleased cells; CD34^bright^ cells. Consequently, the magnetic (unreleased) cell fraction was enriched with CD34^bright^ cells and nonmagnetic (released) cell fraction was enriched with CD34^high^ cells. For nonmagnetic (released) cell fraction, a third separation was able to be performed to sort PROCR^hi^ and PROCR^lo^ fractions as described above (Figure 6 and Figure 7B). The efficiency of separation was evaluated by flow cytometry. All cell fractions were then assessed for proliferative potential by endothelial colony-forming culture.

### Flow cytometry.

The following anti-mouse Abs conjugated with different fluorochrome were used for flow cytometry sorting and analysis: CD31 (clone 390), CD45 (clone 30-F11), and Ter119 (clone TER-119) (all above Abs were purchased from eBioscience). For human cell flow cytometry analysis, the following anti-human Abs were used: CD31 (BD Pharmingen or eBioscience, clone WM59), CD34 (BioLegend, clone 561), CD45 (eBioscience or BioLegend, clone 2D1; BD, clone HI30), CD235a (BioLegend, clone HI264), and CD201 (EPCR, PROCR) (Miltenyi Biotec, clone REA337). Cell analysis and sorting were performed on LSR4, LSRII, FACSCantoII, FACSAria, and SORPAria flow cytometers (BD Biosciences). FlowJo software (BD Pharmingen) was used to analyze flow cytometry data.

For human quantitative PCR (qPCR) (Figure 8A), methylcellulose colony assay (MCA) (Supplemental Figure 5C), EC colony-forming assay (Supplemental Figure 5D), and magnetically CD34-enriched fresh CBMCs (CD34 MicroBead Kit, Ultrapure, human, 130-100-453, Miltenyi Biotec, according to the manufacture’s protocol) were incubated with anti-CD201 (EPCR, PROCR) (clone REA337, Miltenyi Biotec) and anti-CD34 Abs (BioLegend, clone 561) and sorted into 4 fractions: (i) CD34^hi^PROCR^lo^, (ii) CD34^hi^PROCR^hi^, (iii) CD34^bright^PROCR^lo^, and (iv) CD34^bright^PROCR^hi^ performed on FACSAria and used for each experiment. This cell sorting was performed by FACSAria and FACSDiva software (BD Biosciences) was used for the sorting. The flow rate was set at 1.0.

For murine single EC culture, TT^+^ cells were flow sorted and each cell was directly loaded into each well of 96-well plate coated with OP9 stromal cell monolayer (see below). For EC colony-forming limiting dilution assay, 20, 50, or 100 P6 heart TT^+^ ECs, or 200, 500, and 1,000 P6 lung TT^+^ ECs were sorted into individual wells of OP9-coated 96-well plates, respectively.

### EC formation culture.

For murine EC culture, OP9 stromal cells were maintained in OP9 medium (alpha-MEM medium, Gibco; with 20% FBS, Hyclone; and 0.5% penicillin/streptomycin, Gibco). To culture endothelial colonies, isolated murine ECs were resuspended in EC culture medium (alpha-MEM with 10% FBS, 5 × 10^−5^ M β-mercaptoethanol, Sigma; and 0.5% penicillin/streptomycin, Gibco). After 24 hours, nonadherent cells were removed by changing fresh medium. Medium was changed every 3 days afterward until use. For human EC culture, the cells were resuspended in complete medium (Endothelial Cell Growth Medium-2, Lonza) with 10% FBS (Hyclone) and replated on 0.1% type I rat tail collagen–coated (Corning) tissue culture plates.

### MCA.

MCA was performed using MethoCult (H4434, StemCell Technologies) according to the manufacturer’s protocol.

### A 3D in vitro tube-forming assay in collagen gel.

200 Pa stiffness pig skin type I collagen gels were made according to the manufacturer’s instructions (Standardized Oligomer Polymerization Kit; Geniphys). Human platelet lysate (10%) (Sexton Biotechnologies) was then added into the gel and the liquid gel was kept on ice. Human ECs were resuspended in the gel at a density of ~1 × 10^6^ cells/1 mL gel and each 50 μL gel was transferred to a well in a 96-well plate. The cellularized gels were incubated at 37°C for 30 minutes to solidify. Next, the gels were covered by adding 100 μL complete EGM2 medium to the wells. The cultures were checked under a microscope every 12 hours until lumenized vessel-like structures were formed and identified.

### In vivo gel implantation.

Blood from TAM injected Tie2cre ROSATT pups were cultured on OP9 for 3–8 weeks. Cultured cells from individual pups were resuspended in 250 μL 200 Pa collagen gel (Geniphys, Standardized Oligomer Polymerization Kit) plus 10% human platelet lysate (Cook) on ice and transferred into 1 well of 48-well plate. The gel was placed in a 37°C incubator to polymerize for 30 minutes. Next the cellularized gels were transplanted s.c. into the flanks of 6- to 12-week-old NOD/SCID mice. The gels were retrieved from the animals at various time points between 14 days and 10 months following implantation. To test the vessel-forming potential of uncultured mouse or human circulating ECFCs, blood from 10 P3 Tie2Cre ROSATT pups (TAM injected on P0, P1, and P2), or MACS-isolated 300,000 human CB CD34^+^ cells were each suspended into a single collagen gel.

### Surgeries.

For EC collagen gel transplantation assay, cells were resuspended in 250 μL 200 Pa pig skin type I collagen gel (Geniphys, Standardized Oligomer Polymerization Kit) plus 10% human platelet lysate on ice. When murine ECs were tested, 50 μg/mL murine VEGF (Peprotech) and 100 μg/mL murine FGF2 (Peprotech) were added to the gels. Each cellularized gel was transferred into 1 well in a 48-well plate and incubated at 37°C to polymerize for 30 minutes. Next, the cellularized gels were transplanted s.c. into the flanks of 6- to 12-week-old NOD/SCID mice as previously described (56). The gels were retrieved from the animals at various time points between 14 days and 4 months following implant.

### Cell culture IHC and immunofluorescence staining.

For IHC staining of endothelial colonies on OP9 coculture plates, the cultures were fixed with 4% paraformaldehyde (PFA) for 30 minutes at room temperature (RT). After washing, the samples were blocked with 2% skim milk (Sigma) in 0.1% Triton (Sigma) PBS solution (PBSMT solution) for 30 minutes at RT and then stained with 1:100 rat anti-mouse CD31 (BD Pharmingen, clone MEC 13.3) or rat anti-mouse Flk1 (BD Pharmingen, clone Avas 12α1) Ab in PBSMT at RT for 2 hours or at 4°C overnight. Next the plates were stained with 1:200 alkaline phosphatase-conjugated donkey anti-rat IgG secondary Ab (Jackson ImmunoResearch) in PBSMT at RT for 2 hours or at 4°C overnight. The colonies were visualized by VECTOR Blue Alkaline Phosphatase (Blue AP) Substrate Kit.

For culture immunofluorescence staining of murine EC culture, fixed cultures were blocked with 10% goat serum in 0.5% triton PBS solution (blocking solution) and sequentially stained with primary Ab (1:100 rat anti-mouse CD31, clone MEC 13.3) and secondary Ab (1:200 Alexa Fluor 488- or 647-conjugated goat anti-rat IgG; Cell Signaling Technology) in blocking buffer. All culture pictures were visualized using Leica DM IL microscope with a SPOT RT3 camera (Spot Imaging).

### Tissue immunofluorescent staining.

To visualize TT^+^ vessels in freshly collected muscle or collagen gel samples after transplantation, a Leica mz9.5 stereomicroscope with LEJ eqb 100 isolated lamp power supply was used. To detect the perfusion of implanted vasculature that had inosculated with host vessels, 100 μL fluorescein-conjugated IB4 (Vector Laboratories, for mice vessels) or 100 μL fluorescein-labeled Ulex Europaeus Agglutinin I for human vessels (Vector Laboratories) were intravascularly injected into the mice 30 minutes prior to euthanasia and sample collection. To take confocal images of tissues or transplanted gels, the samples were collected and fixed in 4% PFA at 4°C overnight, rinsed in 30% sucrose o at 4°C overnight, and then mounted in OCT compound (Thermo Fisher Scientific) on dry ice. The tissue blocks were cut into 10–30 μm sections using a Leica CM3050s cryostat and mounted on Superfrost Plus Gold microscope glass slides (Thermo Fisher Scientific). After blocking with blocking buffer at RT for 1 hour, the slides were then stained with different unconjugated primary Abs, including rat anti-mouse CD31 (BD Pharmingen, clone MEC 13.3, 1:100), and rabbit anti-ERG (Abcam, clone EPR3864, 1:100) at 4°C overnight. Then, 1:200 Alexa Fluor 488-, 555-, or 647-conjugated goat anti-rat, anti-rabbit, or anti-mouse IgG Abs (Cell Signaling Technology) were used for secondary staining at 4°C overnight. For some stainings, the following conjugated Abs were used: Alexa Fluor 647–conjugated mouse anti-human CD31 (BD Pharmingen, clone WM59, 1:50) and Alexa Fluor 488– or 594–conjugated mouse anti–smooth muscle actin–α (eBioscience, clone 1A4, 1:100). After staining, the samples were mounted with ProLong Gold Antifade Mountant with DAPI (Molecular Probes) and *Z*-stack confocal images were taken on Olympus FV1000 microscope.

All fluorescent pictures were processed using ImageJ (NIH) software to produce merge images. The 3D reconstruction of CD31^+^ and TT^+^ vessels in tissues or gels was performed using Imaris software. The volumes of blood vessels were calculated by Imaris software using the Surface function according to the manufacturer’s instruction.

### qPCR.

RNA from each sample was extracted using RNeasy Plus Micro kit (Qiagen). Reverse transcription was done using SuperScript IV Reverse Transcriptase (Thermo Fisher Scientific). qPCR was performed on StepOnePlus (Thermo Fisher Scientific) with PowerUp SYBR Green Master Mix (Applied Biosystems). β-Actin was used as a reference gene to calculate transcript abundance of each target gene. The expression level fold-change between sample genes and reference genes was calculated by standard curve, where for every run, a new standard curve was constructed.

### Statistics.

All data are presented as the mean ± SD or SEM. Unpaired 2-tailed Student’s *t* test to compare 2 groups or Tukey-Kramer post hoc test to compare more than 2 groups were used to determine significance. A *P* value greater than 0.05 was considered nonsignificant, while a *P* value less than 0.05 was considered significant and marked *P < 0.05, **P < 0.01. All statistical analyses were calculated using GraphPad Prism or Microsoft Excel software.

### Study approval.

All animal experiments were conducted in accordance with the *Guidelines for the Care and Use of Laboratory Animals* (National Academies Press, 2011), and all protocols were approved by the IACUC of the Indiana University School of Medicine. Human umbilical cords and umbilical CB samples destined to be discarded (nonidentified surgical waste and not considered human research) were collected immediately after cesarean section in Indiana University Health Methodist Hospital. Human umbilical CB samples were also collected in Nara Medical University after receipt of written informed consent, approved by Nara Medical University Ethics Committee (G151).

## Author contributions

The study was conceptualized by YL, KB, CHG, and MCY. The methodology was designed by YL, KB, CHG, WCS, MY, NP, SPT, XX, RHA, KN, and JY. The investigation was conducted by KB, JM, and TH. Data curation was performed by HG. The manuscript was written by YL, KB, CHG, and MCY. The study was supervised by YL, KB, KH, and MCY. Funding was acquired by YL, KB, DPB, MPM, KH, and MCY.

## Supplementary Material

Supplemental data

## Figures and Tables

**Table 1:**
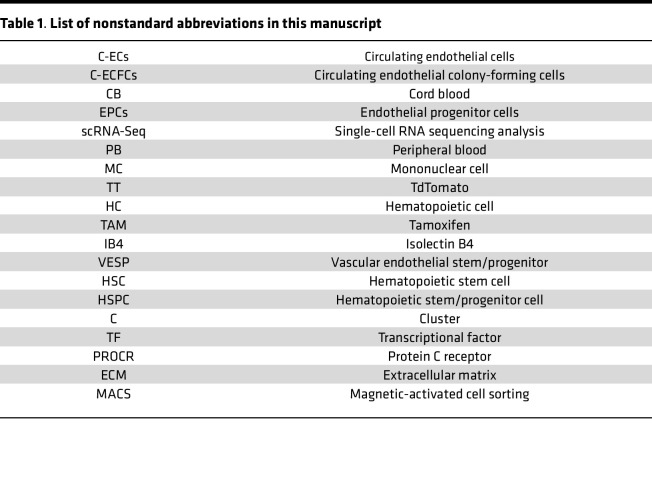
List of nonstandard abbreviations in this manuscript

## Version 1 01/24/2023

In-Press Preview

## Version 2 03/08/2023

Electronic publication
