# Supplementary material for: Origin, prospective identification, and function of circulating endothelial colony-forming cells in mice and humans
Source: JCI Insight. 2023 Mar 8;8(5):e164781. doi: 10.1172/jci.insight.164781 (PMC10077473; doi:10.1172/jci.insight.164781)
Supplement: Supplemental data [file jciinsight-8-164781-s222.pdf]

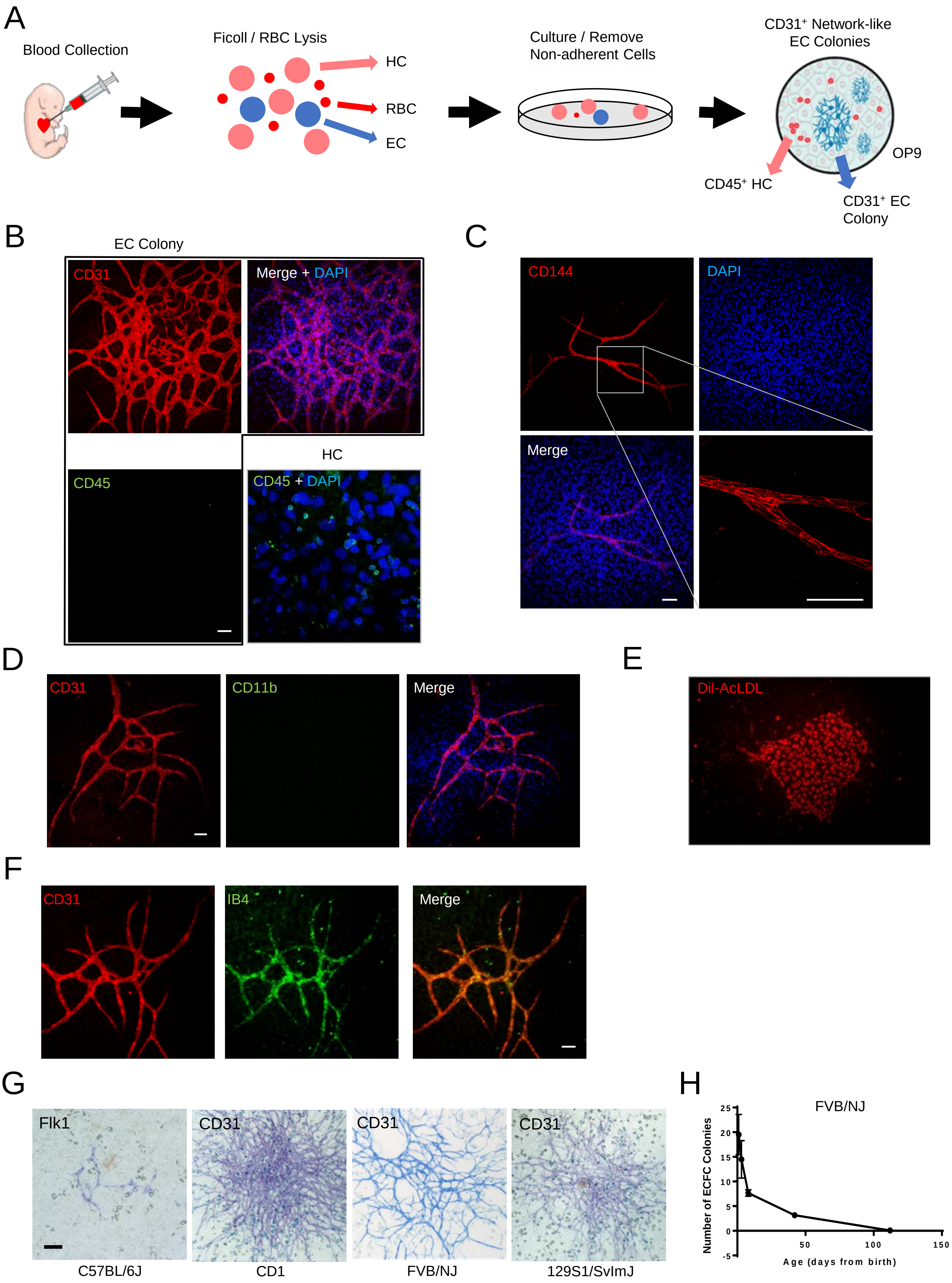

A
Blood Collection
Ficoll / RBC Lysis
HC
RBC
EC
Culture / Remove Non-adherent Cells
CD31+ Network-like EC Colonies
OP9
CD45+ HC
CD31+ EC Colony
B
EC Colony
CD31
Merge + DAPI
HC
CD45
CD45 + DAPI
C
CD144
DAPI
Merge
D
CD31
CD11b
Merge
E
Dil-AcLDL
F
CD31
IB4
Merge
G
Flk1
CD31
CD31
CD31
C57BL/6J
CD1
FVB/NJ
129S1/SvImJ
H
FVB/NJ
Number of ECFC Colonies
Age (days from birth)
25
20
15
10
5
0
-5
50
100
150

Supplemental Figure 1. Neonatal/juvenile murine peripheral blood contains circulating endothelial colony forming cells (C-ECFCs).

(A) Schematic of cultured C-ECFC from murine neonatal/juvenile peripheral blood. RBC, red blood cells; HC, hematopoietic cells; EC, endothelial cells. (B) Murine C-ECFC derived EC colonies express the endothelial marker CD31 (red), but not hematopoietic marker CD45 (green). Scale, 100 μm. (C) Murine C-ECFC derived EC colonies express the endothelial marker CD144 (red). Scale, 100 μm. (D) Murine C-ECFC derived EC colonies do not express the myeloid hematopoietic marker CD11b (green). Scale, 100 μm. (E) Murine C-ECFC derived EC colonies can ingest AcLDL (red). Dil-AcLDL, Dil-Acetylated Low Density Lipoprotein. (F) Murine C-ECFC derived EC colonies can bind lectin (green). IB4, isolectin B4. Scale, 100 μm. (G) Representative Mouse C-ECFC derived colonies from the peripheral blood of C57BL/6J (n>50), CD1 (n=5), FVB/NJ (n=3) and 129S1/SvImJ (n>50). Pictures show the staining of alkaline phosphatase conjugated anti-rat IgG secondary antibodies against rat anti-mouse Flk1 (left), or CD31 (other 3 panels). Scale, 200 μm. (H) Kinetics of emergence of C-ECFC derived EC colonies in FVB/NJ mice blood. Three to 4 mice per time point.

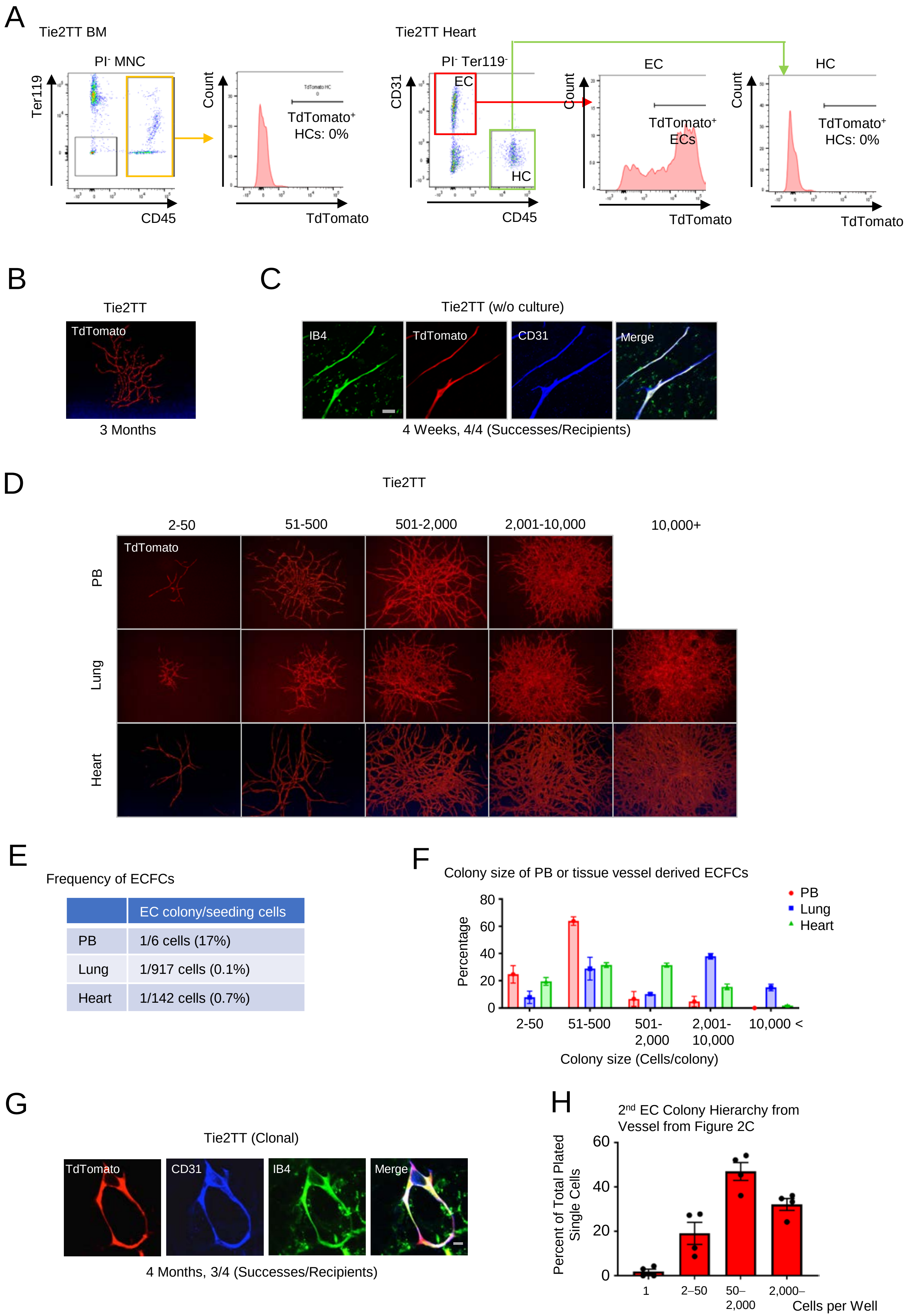

	EC colony/seeding cells
PB	1/6 cells (17%)
Lung	1/917 cells (0.1%)
Heart	1/142 cells (0.7%)

Supplemental Figure 2. Murine and human CECs contain endothelial colony forming cell that can form functional blood vessel in vitro and in vivo.

(A) Flow cytometry analysis of postnatal day 4 Tie2TT mice. TdTomato did not label hematopoietic cells (HCs) but labeled endothelial cells (ECs) in the bone marrow and heart. (B) A picture of a TdTomato$^+$ secondary colony from TdTomato$^+$ vessels in the gel of 3 months after transplantation. (C) Four weeks after collagen-plug transplantation using PB of Tie2TT (P2), uncultured CEC derived blood vessels (TdTomato$^+$) are inosculated with host vasculatures (shown by the labeling of isolectin B4). (D) EC colony images according to the number of cells per colony from PB, lung, heart in Tie2TT mouse (P6). From left to right: 2-50, 51-500, 501-2000, 2001-10000, >10000 cells per colony are shown. Note that colonies of >10000 cells can only be detected from lung and heart. (E) Table showing frequency of appearance of EC colonies per seeded cells (CD34$^+$CD45$^-$ TdTomato$^+$ fraction); EC colonies from PB are more frequent than from lung or heart. (F) Bar graph showing the percentage of cells per EC colony; PB-derived EC colonies have fewer cells per colony. Data are means±SEM; Colonies were counted in 3-4 independent experiments. (G) “Single Tie2CreTT TT$^+$ cell derived EC colony”-derived blood vessel in vivo 4 months after transplantation. Three successes out of 4 recipients. (H) 2nd EC culture grew from human cord blood CEC derived blood vessels show a hierarchy of

proliferative potential in single cell colony forming assay. Data are means ± SEM; Colonies were counted in 4 independent experiments.

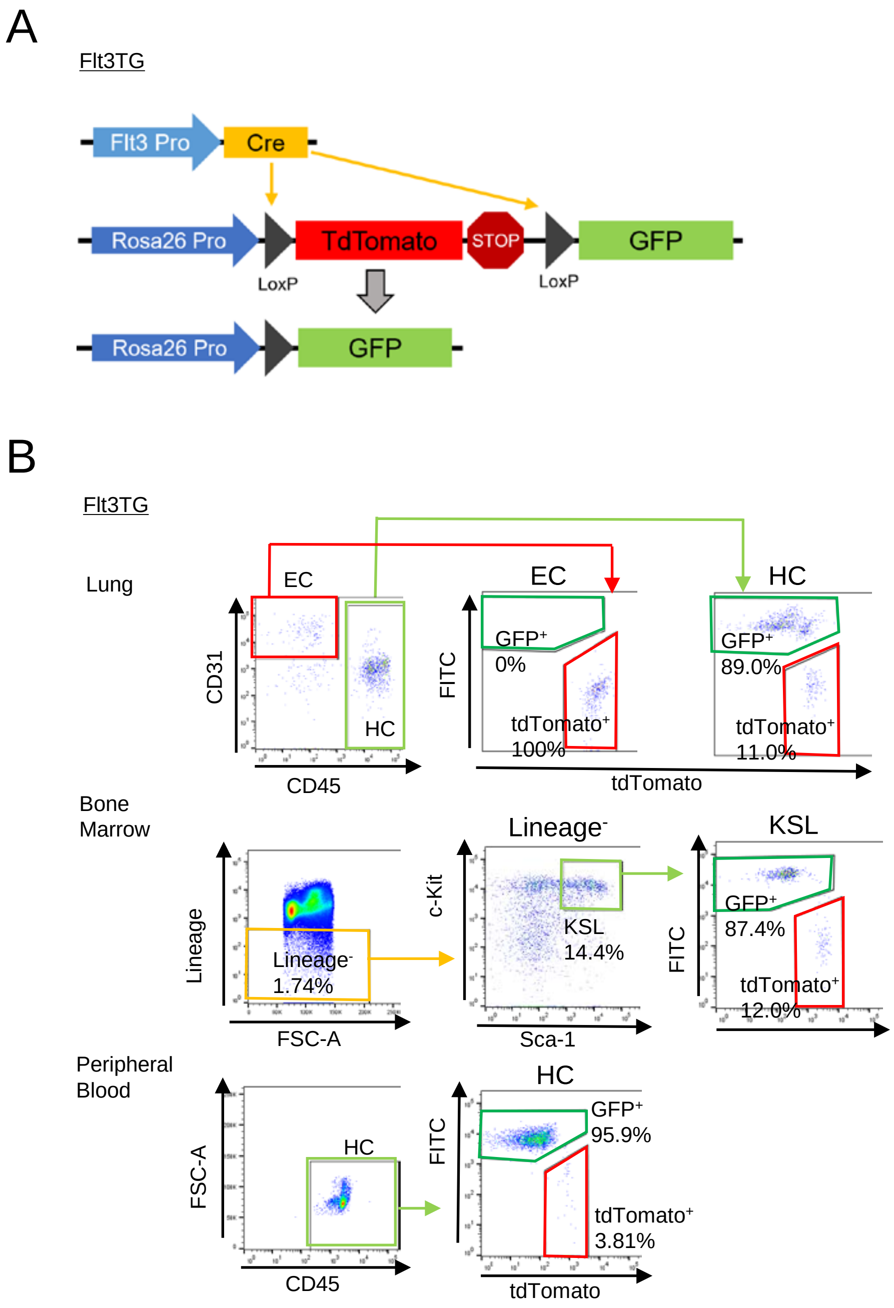

A
Flt3TG
Flt3 Pro
Cre
Rosa26 Pro
TdTomato
STOP
GFP
LoxP
LoxP
Rosa26 Pro
GFP
B
Flt3TG
Lung
EC
HC
CD31
CD45
EC
HC
FITC
GFP+
0%
tdTomato+
100%
GFP+
89.0%
tdTomato+
11.0%
tdTomato
Bone
Marrow
Lineage
FSC-A
Lineage-
1.74%
Lineage-
c-Kit
Sca-1
KSL
14.4%
KSL
FITC
GFP+
87.4%
tdTomato+
12.0%
Peripheral
Blood
FSC-A
CD45
HC
HC
FITC
GFP+
95.9%
tdTomato+
3.81%
tdTomato

Supplemental Figure 3. Schematic and flow cytometry result of Flt3mTmG mouse for investigating the origin of circulating ECFCs.

(A) Schematic of Cre-loxP recombination in Flt3Cre;mTmG mouse. Cre recombinase is expressed under the control of Flt3 regulatory elements. Cre-mediated excision of TdTomato results in induction of GFP expression. (B) Flow cytometry analysis of postnatal day 14 Flt3Cre:mTmG mice. GFP did not label EC in the lung but labeled almost all hematopoietic cells and hematopoietic stem cells in the lung, bone marrow and peripheral blood.

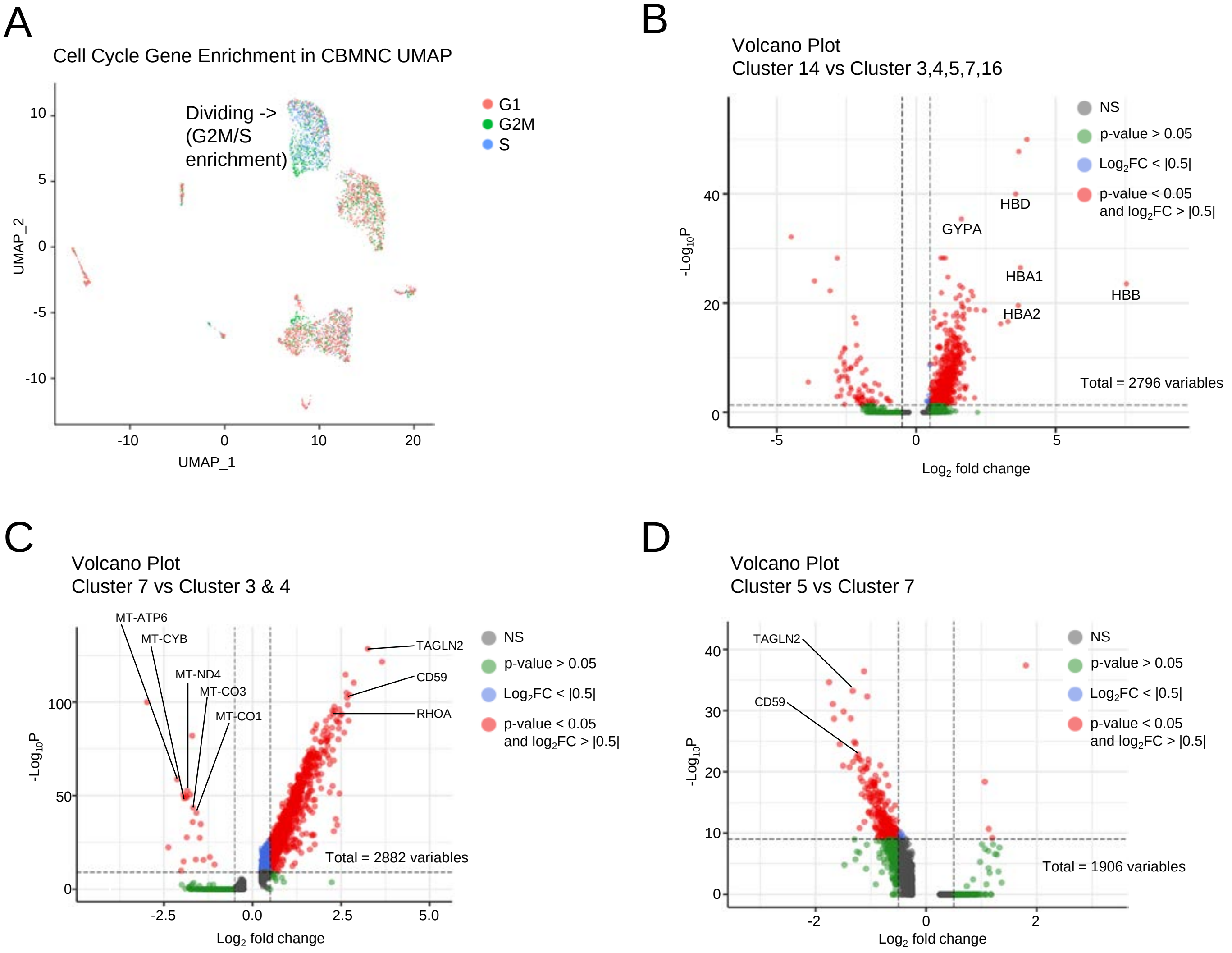
A
Cell Cycle Gene Enrichment in CBMNC UMAP
Dividing -> (G2M/S enrichment)
G1
G2M
S
UMAP_1
UMAP_2
B
Volcano Plot
Cluster 14 vs Cluster 3,4,5,7,16
NS
p-value > 0.05
Log2FC < |0.5|
p-value < 0.05 and log2FC > |0.5|
HBD
GYPA
HBA1
HBB
HBA2
Total = 2796 variables
Log2 fold change
-Log10P
C
Volcano Plot
Cluster 7 vs Cluster 3 & 4
MT-ATP6
MT-CYB
MT-ND4
MT-CO3
MT-CO1
TAGLN2
CD59
RHOA
Total = 2882 variables
NS
p-value > 0.05
Log2FC < |0.5|
p-value < 0.05 and log2FC > |0.5|
Log2 fold change
-Log10P
D
Volcano Plot
Cluster 5 vs Cluster 7
TAGLN2
CD59
NS
p-value > 0.05
Log2FC < |0.5|
p-value < 0.05 and log2FC > |0.5|
Total = 1906 variables
Log2 fold change
-Log10P

Supplemental Figure 4. Single cell RNA sequencing reveals unique endothelial cell related clusters in circulating human cord blood mononuclear cells.

(A) Cell cycle gene enrichment analysis in UMAP plots of CBMNCs. G2M and S phase specific genes are enriched in cluster 1, 2, and 6 of CBMNCs. (B) Volcano plot comparing cluster 14 vs cluster 3, 4, 5, 7, and 16. Dots are colored based on log2 fold change and p-value. (C) Volcano plot comparing cluster 7 vs cluster 3 &4. (D) Volcano plot comparing cluster 5 and cluster 7.

A

Flow Cytometry of EasySep-Sorted Cells by APC Beads

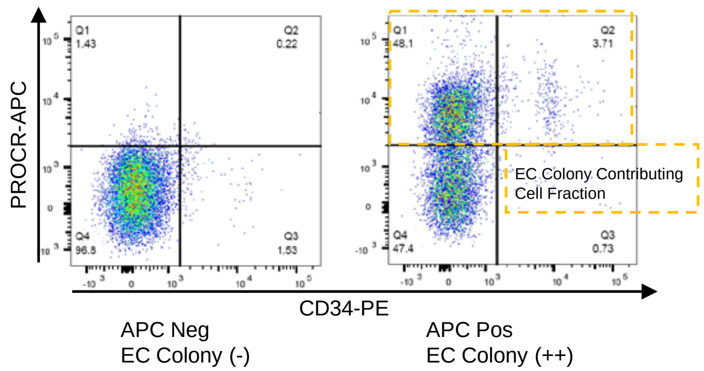

B

Flow Cytometry of MACS-Sorted Cells by CD34 Beads

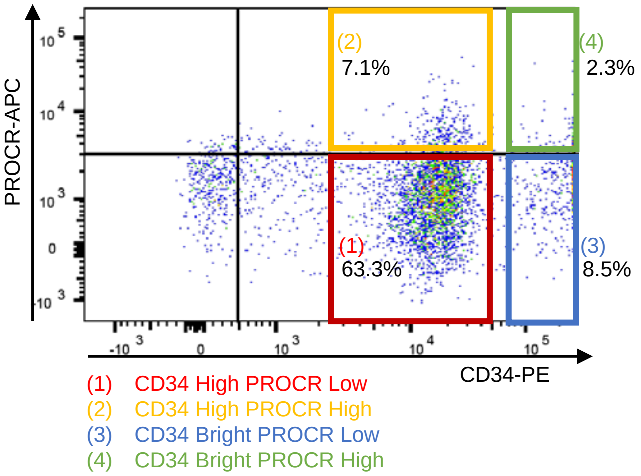

C

Methylcellulose Assay

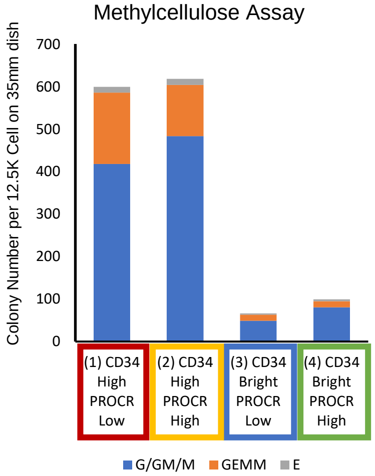

D

In vitro EC Colony-Forming Assay after FACS Sorting

	Exp.1	Exp.2	Exp.3
(1) CD34 High PROCR Low (216K,175K,268K in each Exp)	0	0	0
(2) CD34 High PROCR High (38K,27K,80K in each Exp)	0	0	0
(3) CD34 Bright PROCR Low (10K,27K,23K in each Exp)	0	0	0
(4) CD34 Bright PROCR High (10K,27k,23K in each Exp)	3 (ECFC)	0	0

E

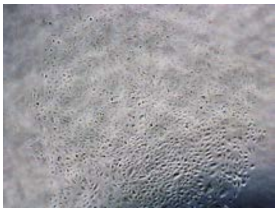

Representative EC Colony (from Exp.1 of Supplemental Figure 5D)

Supplemental Figure 5. Colony-forming potential of PROCR-high and CD34-bright endothelial population in CBMNC identifies C-ECFCs within CECs.

(A) Representative flow cytometry analysis of EasySep-sorted CBMNCs by PROCR-APC and anti-APC beads. Cells from APC negative fraction do not yield EC colonies, whereas those from APC positive fraction yield EC colonies. (B) Representative flow cytometry analysis of MACS-sorted CBMNCs by CD34 beads. CD34 positive cells can be divided into 4 fractions using CD34 and PROCR expression levels; (1) CD34 High PROCR High (in red), (2) CD34 High PROCR Low (in orange), (3) CD34 Bright (= very high) PROCR Low (in blue), and (4) CD34 Bright PROCR High (in green). CD34 bright cells represent about 1/8 of total CD34 positive cells. (C) Hematopoietic colony number after methylcellulose assay using cells from 4 fractions sorted by FACS. CD34 high fractions ((1) and (2)) give rise to 6-10 times more hematopoietic colonies compared with CD34 bright fractions ((3) and (4)). (D) Colony numbers after in vitro EC colony-forming assay from 4 fractions sorted by FACS. Only CD34 bright PROCR high fractions (4) yielded EC colonies once. Other fractions did not yield EC colonies. (E) Representative EC colony (about 1,000 cells per colony) with cobblestone appearance from CD34 bright PROCR high fraction (4).

Supplemental Table 1. Enriched Regulons by SCENIC Analysis

Cluster 16 vs cluster 3, 4, 5, and 7.

Regulon (gene number)	Enriched Cluster(s)
GABPA (1352)	16
CREB3 (15)	16
MAZ_extended (2768)	16
NR2F2_extended (10)	16
FOXC2_extended (77)	16
ZNF358_extended (649)	16
ATF4 (16)	16
YY1 (1233)	16
NFIC_extended (14)	16
HOXB2_extended (23)	16
CUX1 (12)	16
MAX_extended (2634)	16
FOXC1_extended (24)	16
NR4A1_extended (16)	16
ZNF664 (39)	16

NFKB2 (72)	3_4_5_7
XBP1 (237)	3_4_5_7
JUND (38)	3_4_5_7
CREM (43)	3_4_5_7
TAF7 (14)	3_4_5_7
BCL3_extended (78)	3_4_5_7
IRF1 (579)	3_4_5_7
ATF3 (2030)	3_4_5_7
KLF3_extended (126)	3_4_5_7
KLF2_extended (92)	3_4_5_7
JUNB (21)	3_4_5_7
KLF10 (41)	3_4_5_7
ATF5 (50)	3_4_5_7
JUN (29)	3_4_5_7
KLF4 (236)	3_4_5_7

Supplemental Table 1. Enriched Regulons by SCENIC Analysis

Cluster 14 vs cluster 3, 4, 5, and 7.

Regulon (gene number)	Enriched Cluster(s)
REST_extended (657)	14
YY1 (2148)	14
SRF (28)	14
HDAC2_extended (1631)	14
MAFF_extended (12)	14
MXD3_extended (49)	14
BHLHE40_extended (1970)	14
SAP30_extended (452)	14
NFIL3 (29)	14
TCF3 (35)	14
HOXA10 (13)	14
MAZ_extended (2751)	14
NFYB (17)	14
THAP11 (160)	14
E2F4 (24)	14
RAD21_extended (2471)	14
TFDP1_extended (70)	14
ZNF664 (38)	14
HMGN3_extended (11)	14
TFDP2_extended (35)	14
TBP_extended (33)	14
MAX_extended (2734)	14
POLE3_extended (757)	14

NFE2L2 (27)	3_4_5_7
IRF1 (659)	3_4_5_7
CREB3_extended (541)	3_4_5_7
ATF3 (2460)	3_4_5_7
JUND (41)	3_4_5_7
TAF7 (28)	3_4_5_7
XBP1 (192)	3_4_5_7
ATF4 (40)	3_4_5_7

JUNB (24)	3_4_5_7
ATF5 (136)	3_4_5_7
KLF10 (94)	3_4_5_7
KLF2_extended (43)	3_4_5_7
KLF4 (258)	3_4_5_7
JUN (29)	3_4_5_7
NFKB2 (102)	3_4_5_7
BCL3 (18)	3_4_5_7
ETS2 (2037)	3_4_5_7
CREM (108)	3_4_5_7
CREB5 (58)	3_4_5_7
CEBPB (50)	3_4_5_7
DDIT3 (55)	3_4_5_7
FOSL2 (25)	3_4_5_7
EGR1_extended (22)	3_4_5_7
MAFB_extended (28)	3_4_5_7
ZNF143_extended (21)	3_4_5_7
GTF2B (11)	3_4_5_7
SOX4_extended (17)	3_4_5_7
IRF7 (175)	3_4_5_7
FOSB_extended (45)	3_4_5_7
FOS_extended (595)	3_4_5_7
POLR2A (1612)	3_4_5_7
IRF9 (94)	3_4_5_7
IRF2 (155)	3_4_5_7
EZH2 (33)	3_4_5_7
FOXK2_extended (48)	3_4_5_7
POU3F1_extended (21)	3_4_5_7
PLAGL1 (12)	3_4_5_7
HOXA13_extended (25)	3_4_5_7
TAL1_extended (195)	3_4_5_7
FOXO1_extended (199)	3_4_5_7
STAT1 (240)	3_4_5_7
THAP1 (575)	3_4_5_7
HIST1H2BN_extended (683)	3_4_5_7

KDM5B_extended (854)	3_4_5_7
MYC_extended (2754)	3_4_5_7
SP3_extended (1034)	3_4_5_7
RCOR1_extended (724)	3_4_5_7
MAFK_extended (1157)	3_4_5_7
ELK4_extended (1112)	3_4_5_7
ESRRA_extended (2139)	3_4_5_7
ZNF274_extended (1448)	3_4_5_7
JDP2 (34)	3_4_5_7
ATF6_extended (182)	3_4_5_7
ZBTB7A_extended (162)	3_4_5_7
STAT2 (314)	3_4_5_7
FOXO3_extended (81)	3_4_5_7
SPI1 (257)	3_4_5_7
EP300 (98)	3_4_5_7
FOXP1 (87)	3_4_5_7
FOXC2_extended (178)	3_4_5_7
ZNF358 (435)	3_4_5_7
ZNF768 (288)	3_4_5_7
ZNF91_extended (161)	3_4_5_7
GABPB1 (443)	3_4_5_7
USF1_extended (494)	3_4_5_7
E2F6_extended (471)	3_4_5_7
ATF6B_extended (255)	3_4_5_7
GATA2 (15)	3_4_5_7
MXI1 (372)	3_4_5_7
ELF2 (830)	3_4_5_7
BCLAF1_extended (2559)	3_4_5_7
KDM5A_extended (1564)	3_4_5_7
IRF3_extended (1223)	3_4_5_7
SREBF2_extended (844)	3_4_5_7
TAF1 (639)	3_4_5_7
SREBF1 (64)	3_4_5_7
CREB1 (12)	3_4_5_7
SMARCA4_extended (1182)	3_4_5_7

HCFC1_extended (1143)	3_4_5_7
GABPA (1291)	3_4_5_7
CTCF_extended (996)	3_4_5_7
ELF4_extended (618)	3_4_5_7
ETV6 (31)	3_4_5_7
PML (1286)	3_4_5_7
GTF2F1_extended (1461)	3_4_5_7
FLI1 (12)	3_4_5_7
ERG_extended (1390)	3_4_5_7
HDAC6_extended (432)	3_4_5_7
ETV3 (894)	3_4_5_7
RELA (540)	3_4_5_7
ZMIZ1_extended (183)	3_4_5_7
NR3C1 (560)	3_4_5_7
ETS1 (1844)	3_4_5_7
ELK3 (2129)	3_4_5_7
CEBPZ_extended (1518)	3_4_5_7
ELF1 (1897)	3_4_5_7
ZEB1_extended (136)	3_4_5_7
SP1 (451)	3_4_5_7
PATZ1_extended (19)	3_4_5_7
FOXC1 (11)	3_4_5_7
TGIF2 (12)	3_4_5_7
NFIA (10)	3_4_5_7
ARID3A (23)	3_4_5_7
MEF2C_extended (11)	3_4_5_7
SNAI1 (10)	3_4_5_7
MAFG_extended (11)	3_4_5_7
KLF11_extended (15)	3_4_5_7
RORA_extended (11)	3_4_5_7
NR2F6 (12)	3_4_5_7
TGIF1 (19)	3_4_5_7
NFKB1 (13)	3_4_5_7
FOXN2 (24)	3_4_5_7
CEBPG (11)	3_4_5_7

PRDM1_extended (13)	3_4_5_7
MLX_extended (12)	3_4_5_7
ATF7_extended (22)	3_4_5_7
KLF3_extended (58)	3_4_5_7
KLF13_extended (31)	3_4_5_7
NR1H2_extended (25)	3_4_5_7
NR4A1_extended (12)	3_4_5_7
ZNF335 (30)	3_4_5_7
MEF2A_extended (17)	3_4_5_7
NR2F2_extended (31)	3_4_5_7
KLF9_extended (25)	3_4_5_7
TFE3_extended (34)	3_4_5_7
TBL1XR1 (12)	3_4_5_7
MEF2D_extended (12)	3_4_5_7
ARNT (43)	3_4_5_7
RFX3_extended (17)	3_4_5_7
ERF (16)	3_4_5_7
RXRA_extended (31)	3_4_5_7
MXD4_extended (68)	3_4_5_7
NFATC2_extended (25)	3_4_5_7
ZNF121 (70)	3_4_5_7
TCF12_extended (20)	3_4_5_7
TEAD2_extended (11)	3_4_5_7
ATF2_extended (25)	3_4_5_7
TBX2_extended (19)	3_4_5_7
FOXJ3_extended (18)	3_4_5_7
UBTF (17)	3_4_5_7
UBP1_extended (40)	3_4_5_7
NR1H3_extended (10)	3_4_5_7
ZNF134 (29)	3_4_5_7
PBX1 (15)	3_4_5_7
STAT3_extended (11)	3_4_5_7
RARA_extended (26)	3_4_5_7
KAT2A_extended (11)	3_4_5_7
SP6_extended (19)	3_4_5_7

SMAD3_extended (11)	3_4_5_7
ZNF493 (16)	3_4_5_7
NFYC_extended (38)	3_4_5_7
NR2C2_extended (13)	3_4_5_7
HIVEP2_extended (29)	3_4_5_7
ZNF217_extended (11)	3_4_5_7
BACH1 (13)	3_4_5_7
AHR (14)	3_4_5_7

Supplemental Table 1. Enriched Regulons by SCENIC Analysis.

Significantly enriched regulons in indicated clusters were shown.

Supplemental Table 2. In vitro EC Colony-Forming Assay Results after Magnetic Sorting

MACS or EasySep Sort PROCR-APC & Anti-APC Beads

	Exp.1	Exp.2	Exp.3
PROCR-APC High (MACS) (100K in every Exp)	9	18	30
PROCR-APC Low (MACS) (1M, 1M, 500K in each Exp)	0	0	0
PROCR-APC High (EasySep) (250K in every Exp)	2	13	Not Performed
PROCR-APC Low (EasySep) (1M, 500K in each Exp)	0	0	Not Performed

MACS CD34 MultiSort Beads > Beads Releasing Reagent > MACS PROCR-APC & Anti-APC Beads

	Exp.1	Exp.2	Exp.3	Exp.4
CD34 Positive > Released > PROCR-APC High (200K, 75K, 250K, 140K in each Exp)	0	0	0	0
CD34 Positive > Released > PROCR-APC Low (400K, 300K, 500K, 280K in each Exp)	0	0	0	0
CD34 Positive > Unreleased (75K, 125K, 70K in each Exp)	Not Performed	4	20	18

Supplemental Table 2. In vitro EC Colony Forming Assay Results after FACS Sorting.
EC colony number is shown in each experiment (Exp). Magnetic sorting with MACS or EasySep is performed before seeding cells on collagen-coated 24 well plate. Detailed magnetic sorting procedures are indicated in Figure 4A. Indicated cell number with parentheses in order was seeded and colonies were evaluated after 2-week culture with EGM-2. Only HPP (high proliferative-potential)-, LPP (low proliferative-potential)-, and cluster ECFCs were counted on day 7-14.

Supplemental Table 3. Primers

qPCR Primers (Human)

ACTB fwd	CCAACCGCGAGAAGATGA
ACTB rev	CCAGAGGCGTACAGGGATAG
CD34 fwd	TCCAGAGACAACCTTGAAGC
CD34 rev	CTTCTTAAACTCCGCACAGC
CDH5 fwd	AGACCACGCCTCTGTCATGTACCAAATC
CDH5 rev	CACGATCTCATACCTGGCCTGCTTC
FLI1 fwd	AGCGTTAGCAAATGCAGCAAGCTGGT
FLI rev	ATTGCCTCACATGCTCCTGTGTCCA
GATA2 fwd2	AGACGACAACCACCACCTTA
GATA2 rev2	TCCTTCTTCATGGTCAGTGG
MCAM fwd	AACACAGTGGGCGCTATGAA
MCAM rev	AACTCGAGGTCCTGGCTACT
PECAM1 fwd	GGTCAGCAGCATCGTGGTCAACATAAC
PECAM1 rev	TGGAGCAGGACAGGTTCAGTCTTTCA
PROCR fwd	CCAACACCACGATCATTCAG
PROCR rev	ATACCGAGTGCGGTTGTAGG
PROM1 fwd	CCTCTGGTGGGGTATTTCTTT
PROM1 rev	CCAGTTTCCGACTCCTTTTG
PTPRC fwd	TAGGGACACGGCTGACTTCCAGATATGA
PTPRC rev	GTGTTGGGCTTTGCCCTGTCACAAATAC

Genotyping Primers (Mouse)

Cre fwd	CGGTCGATGCAACGAGTGAT
Cre rev	CCACCGTCAGTACGTGAGAT
Rosa fwd	CTGTTCCTGTACGGCATGG
Rosa rev	GGCATTAAAGCAGCGTATCC
